# Synaptic Plasticity in Medial Vestibular Nucleus Neurons: Comparison with Computational Requirements of VOR Adaptation

**DOI:** 10.1371/journal.pone.0013182

**Published:** 2010-10-05

**Authors:** John R. W. Menzies, John Porrill, Mayank Dutia, Paul Dean

**Affiliations:** 1 Centre for Integrative Physiology, School of Biomedical Sciences, University of Edinburgh, Edinburgh, United Kingdom; 2 Department of Psychology, University of Sheffield, Sheffield, United Kingdom; The Research Center of Neurobiology-Neurophysiology of Marseille, France

## Abstract

**Background:**

Vestibulo-ocular reflex (VOR) gain adaptation, a longstanding experimental model of cerebellar learning, utilizes sites of plasticity in both cerebellar cortex and brainstem. However, the mechanisms by which the activity of cortical Purkinje cells may guide synaptic plasticity in brainstem vestibular neurons are unclear. Theoretical analyses indicate that vestibular plasticity should depend upon the correlation between Purkinje cell and vestibular afferent inputs, so that, in gain-down learning for example, increased cortical activity should induce long-term depression (LTD) at vestibular synapses.

**Methodology/Principal Findings:**

Here we expressed this correlational learning rule in its simplest form, as an anti-Hebbian, heterosynaptic spike-timing dependent plasticity interaction between excitatory (vestibular) and inhibitory (floccular) inputs converging on medial vestibular nucleus (MVN) neurons (input-spike-timing dependent plasticity, iSTDP). To test this rule, we stimulated vestibular afferents to evoke EPSCs in rat MVN neurons *in vitro*. Control EPSC recordings were followed by an induction protocol where membrane hyperpolarizing pulses, mimicking IPSPs evoked by flocculus inputs, were paired with single vestibular nerve stimuli. A robust LTD developed at vestibular synapses when the afferent EPSPs coincided with membrane hyperpolarisation, while EPSPs occurring before or after the simulated IPSPs induced no lasting change. Furthermore, the iSTDP rule also successfully predicted the effects of a complex protocol using EPSP trains designed to mimic classical conditioning.

**Conclusions:**

These results, in strong support of theoretical predictions, suggest that the cerebellum alters the strength of vestibular synapses on MVN neurons through hetero-synaptic, anti-Hebbian iSTDP. Since the iSTDP rule does not depend on post-synaptic firing, it suggests a possible mechanism for VOR adaptation without compromising gaze-holding and VOR performance *in vivo*.

## Introduction

Adaptation of the vestibulo-ocular reflex (VOR) has been extensively used to test theories of cerebellar function [1]. One such test has concerned the location of sites of plasticity, because Marr-Albus theories predict a site of plasticity (between parallel fibres and Purkinje cells) in cerebellar cortex [2], [3]. Initial experimental results indicated that, contrary to prediction, the site of VOR plasticity in primates lay in the vestibular nuclei [4]. Subsequent work has led to the conclusion that there are in fact at least two sites of plasticity, one in the floccular region of cerebellar cortex and one in the vestibular nuclei [5]. However this conclusion, while not directly falsifying Marr-Albus theories, leaves unanswered the question of why the complex microcircuit of cerebellar cortex should need an additional site of plasticity in the external and much simpler microcircuit of the vestibular nuclei.

A possible answer to this question has been suggested by a recent computational analysis of VOR adaptation [6]. Although VOR performance is accurate at frequencies up to 25 Hz [7], [8], the proposed error signal for VOR adaptation, namely retinal slip, is delayed by ∼100 ms on its way to the flocculus [9], [10]. This delay implies a low frequency limit to learning, and experimental evidence shows that the VOR can only be trained at frequencies below ∼10 Hz [11]. However, simulations using an adaptive-filter model of cerebellar cortex showed that this limitation may be overcome and high frequency accuracy can be achieved, if a learned value of VOR gain in cerebellar cortex is subsequently transferred to the brainstem, for VOR calibration at high frequencies [6].

**Figure 1:**
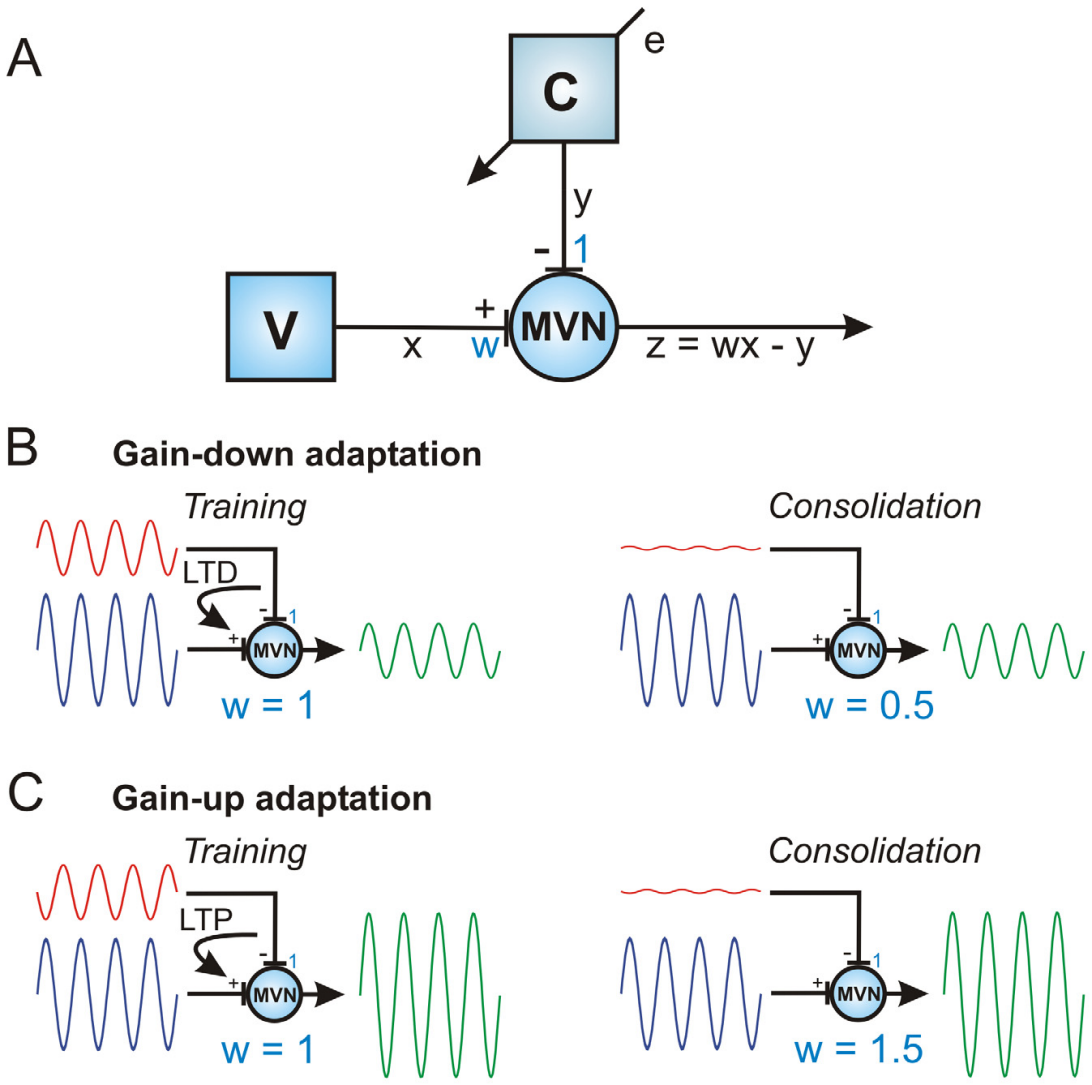
Correlational learning rule for regulation of vestibular synapse strength by cerebellar inhibition: transfer of VOR gain from cerebellar cortex to brainstem. A: Schematic diagram of vestibular and cerebellar inputs to MVN neuron. The input 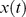 from the vestibular periphery (V) arrives at an excitatory synapse with weight 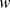. The input 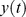 from the cerebellum (C) arrives at an inhibitory synapse with nominal weight 1. The MVN output is treated as the linear combination 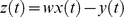 of these two inputs. The input 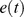 to the cerebellum denotes the retinal-slip training signal assumed to mediate learning in cerebellar cortex. B: Illustration of situation for gain down learning. The brainstem gain 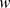 is too high so that the vestibular input on its own would produce an over-large vestibulo-ocular response. During training the cerebellum has learnt to produce an inhibitory input modulated in phase with the vestibular input, leading to cancellation which produces a 50% gain decrease (since 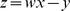 and 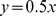, a weight 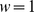 requires 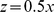). The same result could be produced without the need for cerebellar input if the vestibular afferent synaptic strength (represented by the weight 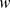) was reduced during consolidation to half its value (since 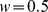 requires 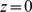). Hence in-phase (positively correlated) inputs to the MVN should drive a long-term depression (LTD) at vestibular afferent synapses during consolidation. C: Illustration of situation for gain up learning. In this case the out-of-phase cerebellar and vestibular afferent inputs to the MVN neurons should drive a long-term potentiation (LTP) of the vestibular afferent synapses during consolidation.

This theoretical analysis provides a rationale for an additional brainstem site of plasticity, and also suggests a learning rule by which the flocculus may regulate the strength of vestibular afferent inputs to brainstem neurons, to alter the gain of the VOR (Fig. 1; cf. [4], [12]). A positive correlation between excitatory vestibular inputs and inhibitory Purkinje cell inputs converging on vestibular neurons should induce long-term depression (LTD) of the vestibular synapses, while a negative correlation should induce long-term potentiation (LTP; Fig. 1). The strength of the vestibular synapses is gradually adjusted to render the cerebellar modulation of the VOR superfluous, consistent with evidence for a minor contribution of the flocculus to well-adapted VOR gain [5]. In effect, gain-changes learnt by the cortex are transferred to the brainstem.

Although there is evidence for plasticity in the intrinsic excitability of vestibular neurons [13], [14], [15] and their afferent synapses [15], [16], [17], the correlational learning rule has not been directly tested [18]. We therefore compared the plasticity of afferent synapses on medial vestibular nucleus (MVN) neurons with theoretical predictions. To facilitate this comparison, we first expressed the correlational learning rule, which is expressed in terms of firing-rates in an equivalent form that specifies the interaction between single excitatory and inhibitory synaptic inputs converging on a post-synaptic neuron. In this form of the rule synaptic plasticity depends explicitly upon the relative timing of the two inputs, and so can be regarded as a form of spike-timing dependent plasticity (termed here input-spike timing plasticity, or iSTDP, see RESULTS). The predictions of this iSTDP rule were tested by pairing brief membrane hyperpolarisations, simulating inhibitory inputs from Purkinje cells, with excitatory post-synaptic currents (EPSCs) evoked by stimulation of vestibular afferents *in vitro*. We also tested the rule with the more complex induction protocol developed by Pugh and Raman [19], [20] to induce plasticity in deep cerebellar nucleus (DCN) neurons, intended to mimic the pattern of mossy fiber and Purkinje cell inputs to DCN neurons in classical eye blink conditioning.

Our results show that repeated coincidence of EPSCs and membrane hyperpolarisation within a narrow temporal window causes robust LTD of the afferent synapses, strongly supporting the theoretical predictions. The effects of the more complex induction protocol were also well predicted by the iSTDP rule. In contrast to proposed mechanisms for plasticity in the DCN that require the silencing of the postsynaptic cell followed by rebound depolarization, it is possible that in the vestibular nuclei vestibular synapse strength may be modulated by iSTDP interactions, independently of postsynaptic firing. Since MVN neurons are directly involved in VOR execution, this would provide a possible mechanism for VOR adaptation without compromising gaze-holding and VOR performance *in vivo*.

## Materials and Methods

### Modeling

#### Correlational Learning Rule

The general form of the correlational learning rule is illustrated schematically in Fig 1, and follows from the consideration that convergent in-phase inhibitory cerebellar inputs and excitatory vestibular inputs tend to cancel at the level of the MVN neurons. Thus for gain-down adaptation the cerebellar cortex adjusts the output of the MVN neurons, and so the gain of the VOR, by the appropriate firing of cerebellar cortical Purkinje cells in phase with the vestibular afferent input (Fig. 1B, [21]). With learning, the required output from the MVN neurons can be achieved, without a need for continuing cerebellar input, if the synaptic weight of the vestibular input is decreased in proportion to the cerebellar inhibitory modulation (Fig. 1B). Thus a positive correlation between the cerebellar inhibitory input and the vestibular afferent input should lead to the induction of a long-term *decrease* in vestibular synaptic weight. Similarly for gain-up adaptation a negative correlation should lead to a long-term *increase* in vestibular synaptic weight (Fig 1C). This correlational learning rule therefore corresponds to the anti-Hebbian covariance learning rule [22], expressed in terms of the firing rates of the cerebellar and vestibular inputs,
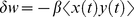
(1)in which weight changes have opposite sign to the correlation of the two inputs (the angle brackets represent a time average over a suitable time scale 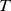). Here 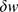 is the change in the weight of the vestibular synapse on the second-order vestibular neuron in the medial vestibular nucleus, 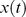 is the difference of the instantaneous firing rate of the vestibular input from its tonic firing rate, and 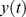 is the difference of the instantaneous firing rate of the Purkinje cell input from its tonic firing rate. The learning rate is fixed by the positive parameter 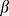. Provided this learning rate is sufficiently slower than the equivalent rate in cerebellar cortex, gain changes learnt in the cortex will be stably transferred to the brainstem [6]. Indirect evidence for the assumption about learning rates comes from studies showing that floccular inactivation only affects VOR gain if a new value has been learnt recently: this evidence is discussed in Porrill and Dean [6].

#### Spike Timing Dependent Plasticity Form of Correlational Learning Rule

In order to experimentally test the above correlational learning rule, we expressed equation (1) in its simplest form as an anti-Hebbian, hetero-synaptic, input-spike-timing dependent plasticity (iSTDP, see RESULTS) rule which defines the interaction between single excitatory and inhibitory synaptic inputs converging on a post-synaptic neuron. Changes in synaptic weight were assumed to be caused by such input pairs, and to depend only on the relative timing of the two input spikes, independently of post-synaptic firing. We will use the term “inter-spike interval” to refer to the interval between spikes in the two separate input streams, and the term “iSTDP profile” for the dependence of vestibular synaptic weight change on the inter-spike interval.

**Figure 2:**
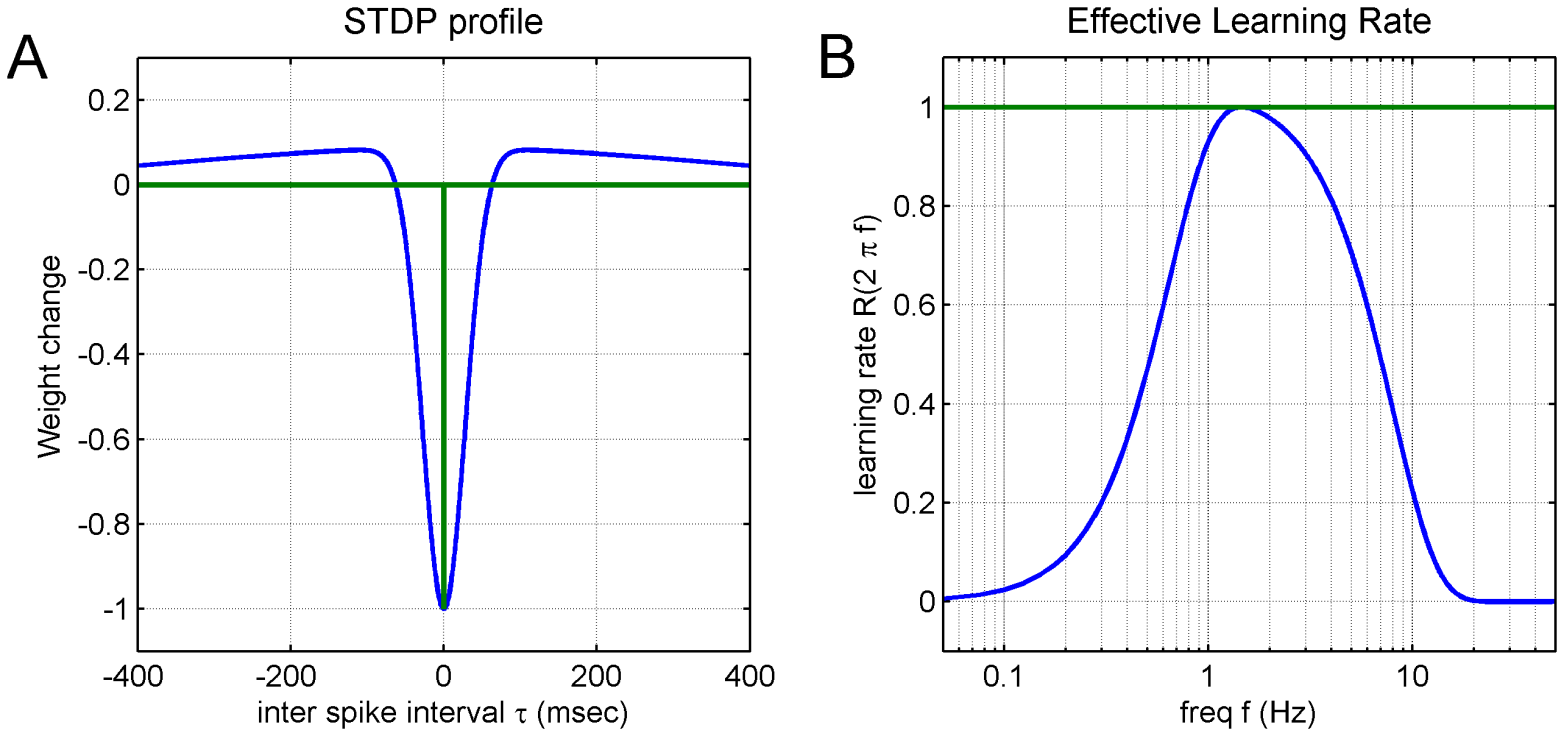
Relation between iSTDP profiles and VOR learning characteristics. Panel A shows two iSTDP profiles, where vestibular synaptic weight change (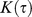 of equation (2) in Methods) is plotted against interval 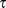 between spikes in the two separate vestibular and cerebellar input streams. Panel B shows the corresponding learning rate functions 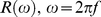 (given by equation (7) in the Methods) plotted against frequency 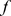 of head motion. The green curve in panel A represents an idealized iSTDP profile with an infinitely narrow and deep LTD dip surrounded by an infinitely wide and shallow LTP plateau. This corresponds to an ‘all-pass’ filter where learning is equally efficient at all frequencies greater than zero, as shown by the corresponding green line in panel B. The blue curve in panels A and B is a filter chosen so that learning is concentrated in the region 0.3–10 Hz as suggested by data for VOR adaptation (see Methods). Its learning rate falls to 20% of maximum at 0.3 and 10 Hz (panel B). The corresponding iSTDP profile (panel A) has a half-width at 20% maximum of 49 ms. The general shape of the iSTDP profile derives in part from the requirement that synaptic weights be stable for tonic or very slowly varying, asynchronous, inputs, which implies that the total area under the profile must be zero (so that the total areas representing synaptic potentiation through LTP, and depression through LTD, must balance).

The iSTDP rule can be derived heuristically by considering the fluctuation in firing rates caused by two additional input spikes separated by an inter-spike interval 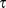 and superimposed on tonic background firing of the cerebellar and vestibular afferent inputs respectively. The two additional spikes produce brief increases in the input firing rates 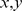 at the time of occurrence of the spikes. If 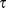 is small enough so that these fluctuations overlap in time, that is they are positively correlated, then by equation (1) this leads to a proportionate decrease in synaptic weight at the vestibular afferent synapse. Thus an important prediction of the iSTDP rule is that there must be a narrow window in which LTD is induced at the vestibular synapse when positively correlated spikes occur in the two input streams, i.e. there is an LTD dip in the iSTDP profile at small values of 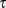. However, because for tonic uncorrelated inputs equation (1) predicts no net overall weight change, the LTD caused by the spike pairs with small 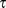 values must be balanced by LTP contributed by spike pairs with large values of 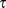; and since such pairs are much more numerous, it follows that the LTP induced by large 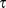 values must be substantially weaker than the LTD induced by small 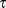 values. The dependence of the change in synaptic weight on 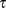 is therefore described by an iSTDP profile
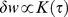
(2)which is characterized by a narrow LTD dip for small values of 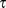, surrounded by weak LTP lobes for larger 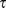 values (see Figure 2).

It is shown below (Frequency-Dependent Form of Correlational Learning Rule, Equation 7 and text) that the learning rate 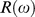 for sinusoidally modulated inputs 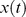, 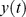 with the same angular frequency 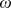 is given by the Fourier transform of the inverted STDP profile 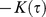 (for more general non-sinusoidal inputs the total weight change is given by a sum over all Fourier components with 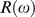 specifying their relative weighting). Hence the form of 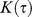 can be constrained by learning rate data for varying head rotation frequency. The exact mathematical form of 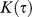 corresponding to equation (1) is an infinitely narrow and deep (delta function) LTD dip surrounded by an infinitely wide and shallow LTP plateau. This has a constant Fourier transform and so corresponds to a learning rule which applies equally well at all frequencies of head movement. However experimental findings show that effective VOR learning is restricted to frequencies below ∼10Hz [10]. As we show below (Frequency-Dependent Form of Correlational Learning Rule) this frequency limit can be incorporated by modifying the form of 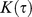, to be a bandpass filter. Nevertheless the essential prediction of the iSTDP rule, of a deep and narrow window where LTD of the vestibular synapse results from the interaction between temporally correlated inhibitory and excitatory input spikes, is not changed when this constraint is taken into account.

#### Application of iSTDP Learning Rule to Spike Trains

The correlational learning rule (2) given in terms of firing rates can be extended to apply to general spiking inputs by taking the sum over all spike pairs of the individual weight changes
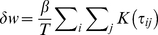
(3)where
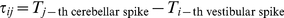
(4)


The iSTDP rule can therefore be used to predict the effects of any arbitrary combination of excitatory and inhibitory input spikes. We exploited this fact to investigate two particular input sequences: (i) the pause-rebound conditioning protocol developed by Pugh and Raman [19] to induce plasticity at mossy-fiber synapses on neurons in the deep cerebellar nuclei, and (ii) simulated *in vivo* learning in MVN neurons with stochastic inputs, to simulate the iSTDP rule superimposed on neuronal spike firing rates similar to those seen *in vivo*. In both cases simulations were implemented in MATLAB™ using the Fast Fourier Transform to implement the convolution in equation (6) (Frequency-Dependent Form of Correlational Learning Rule) efficiently using the convolution theorem [23].

#### Frequency-Dependent Form of Correlational Learning Rule

The general form of the learning rule in equation (1) operates equally effectively over *all* frequencies of head movement, whereas both experimental evidence and theoretical analysis indicate that learning rates in VOR gain adaptation are very markedly affected by head-movement frequency. Indeed it has been argued that only because of these frequency effects is brainstem plasticity required at all [6]. It is therefore important to estimate how far these frequency constraints alter the iSTDP predictions to be used in the present study.

The underlying problem is that, while VOR performance is accurate at frequencies up to at least 20 Hz [7], [8], the retinal slip error signal which drives VOR adaptation is associated with a conduction delay of ∼100 msec in reaching the flocculus [10]. This imposes a theoretical frequency limit above which effective learning cannot occur in the cerebellar cortex of ∼10 Hz (given a plausible eligibility trace), consistent with experimental evidence [11]. For frequencies higher than this limit, VOR calibration can in principle be achieved if the value of VOR gain learnt in the cerebellar cortex for frequencies below ∼10 Hz, is subsequently transferred to the shorter-latency brainstem VOR pathways in the MVN [6]. This is sufficient because the frequency-response of the eye plant above ∼10 Hz is essentially flat (Fig 3B in [6]), so that the value of VOR gain for the highest frequencies which can be effectively learnt in the cortex is also applicable to the shorter-latency pathways through the MVN which mediate the VOR response at higher frequencies. Thus, the correct gain calibration of the brainstem VOR pathways requires a *bandpassed* correlational learning rule, which operates only at frequencies between a lower limit (the frequency at which the plant frequency-response becomes asymptotically flat) and an upper limit (the retinal slip delay-limited maximum frequency at which the cerebellum is able to learn accurately) [6].

**Figure 3:**
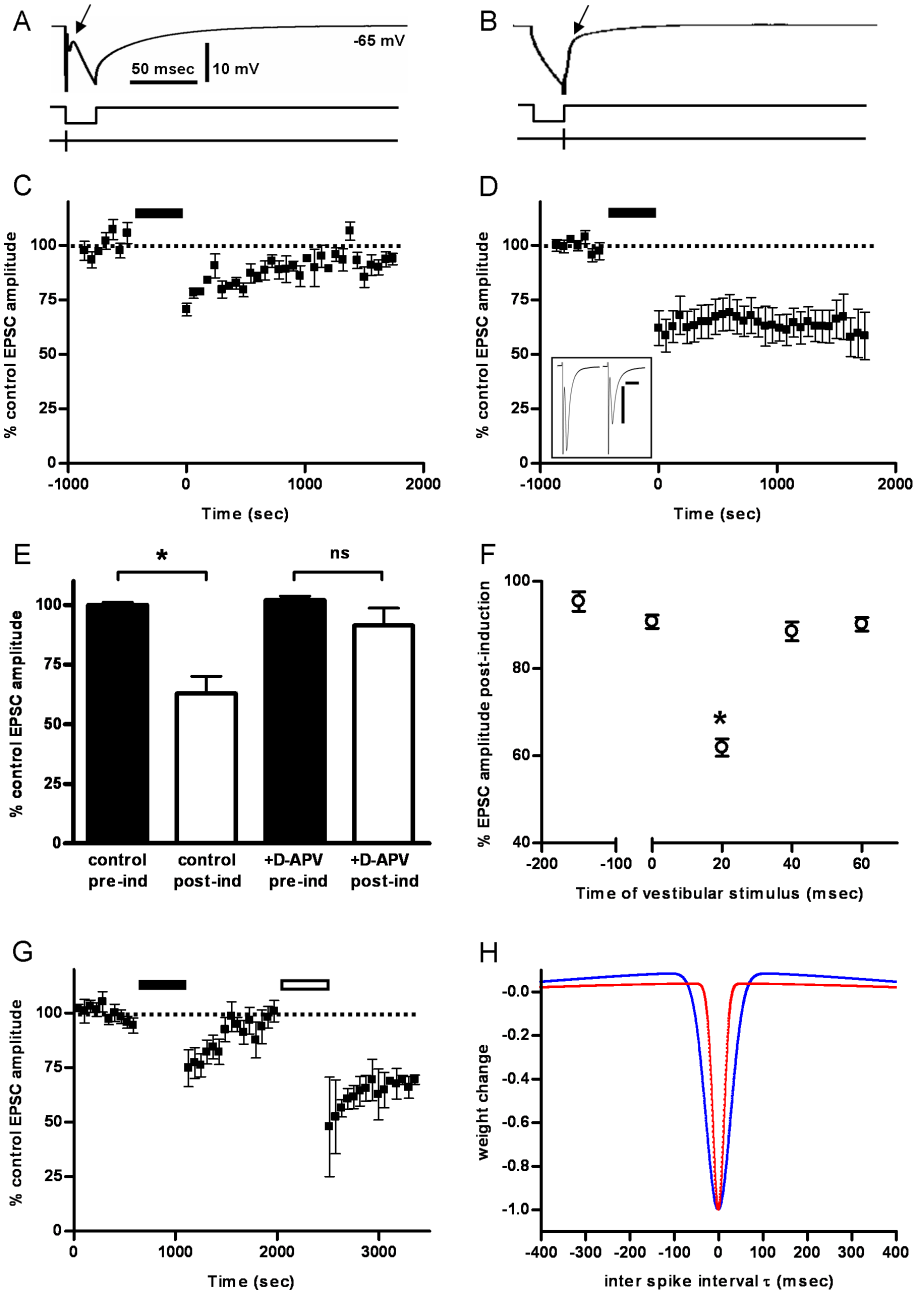
Coincident membrane hyperpolarisation induces long-term depression at the vestibular afferent synapse. A, B, Representation of the iST induction protocols. Single vestibular nerve stimuli were applied at various times relative to a 20 msec hyperpolarizing current pulse (A: T_s_ = 0 msec, vestibular nerve stimulus applied at the start of the membrane hyperpolarisation; B: T_s_ = 20 msec, vestibular stimulus applied to coincide with the maximum membrane hyperpolarisation). The peak of the evoked EPSC is indicated by the arrow in the uppermost records. C, Normalized EPSC amplitude before and after induction with the T_s_ = 0 msec protocol (1000 presentations, every 5 sec, indicated by solid bar). Pairing the vestibular input with the start of the inhibitory input induces a small but significant long-term depression of EPSC amplitude (12.6±0.7% depression; p = 0.01 compared to control, n = 4). D, Normalized EPSC amplitude before and after induction with the T_s_ = 20 msec protocol. Pairing the vestibular input with the peak of the inhibitory input causes a marked long-term depression of vestibular nerve-evoked EPSCs (37±4% depression; p = 0.001 compared to control; n = 9). Inset shows example averaged EPSCs (20 consecutive recordings) before and after a T_s_ = 20 msec induction. Bars indicate 200 pA and 2 msec. The stimulus artefact is truncated. E, Pre-incubation with the NMDA antagonist D-APV (50 µM) prevented the induction of LTD in response to the T_s_ = 20 msec protocol (control, * p = 0.001, n = 9; +D-APV, p = 0.23; n = 4). F, Effects of varying the relative time of the vestibular stimulus on mean EPSC amplitude. The vestibular nerve stimulus was applied at various times with respect to the onset of the inhibitory input (T_s_ = −150 msec, n = 6; T_s_ = 0 msec, n = 4; T_s_ = 20 msec, n = 9; T_s_ = 40 msec, n = 5; or T_s_ = 60 msec, n = 4). Robust LTD is seen when the vestibular input coincides with the peak of the hyperpolarizing input (T_s_ = 20; * p<0.01 compared to T_s_ = −150 msec). G, Three MVN neurons were exposed to two induction protocols in series. In the first induction T_s_ = −150 msec (solid bar) induced a short-term depression which reversed within 15 minutes. Subsequently in the same cell, the T_s_ = 20 msec protocol (open bar) induced a marked significant LTD (32±3% depression; p = 0.04 compared to post-induction with the T_s_ = −150 msec protocol). H. The blue curve is the iSTDP profile constrained by VOR data from Figure 2. The red curve is an iSTDP profile constrained by the experimental data from panel F above. Its half-width is at 20% of maximum is 20 ms.

To obtain different learning rates at different frequencies, the learning rule in equation (1) can be replaced by a more general correlational learning rule of the form
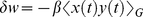
(5)where the expectation 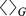 on the right is defined using a convolution kernel 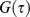


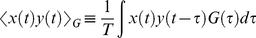
(6)For sinusoidal inputs, 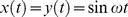 the magnitude of the integral (6) depends on the angular frequency 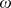, so that different input frequencies have different effective learning rates. The effective learning rate 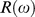 at frequency 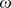 can be calculated analytically (by applying the convolution theorem) as the real part of the Fourier transform of the kernel 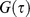 that is
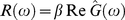
(7)It is clear that the generalized learning rule (5) reduces to the usual covariance rule (1) when the kernel is chosen to be a delta function, and in that case it has equal learning rates at all frequencies (since the Fourier transform of the delta function is a constant).

Applying this generalized rule to spike trains 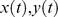 rather than firing rates gives a sum over spikes exactly as in equation (3) with the iSTDP profile being equal to the negative of the convolution kernel : 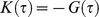. Hence, by choosing an appropriate iSTDP profile 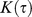, we can shape the frequency response 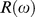 of the learning rule. The requirement that correlational learning should be confined to a restricted frequency range can thus be met by choosing 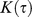 to be an approximate band-pass filter, with upper and lower bandpass limits chosen to correspond to physiologically realistic values (see Results, Fig. 2).

Since the bandpass constraints do not fix the shape of the profile uniquely, we have used a difference of Gaussians (DoG) filter as an example of a time-symmetric filter (DoG filters have been widely used as biologically plausible bandpass filters e.g. [24]). We have also investigated a difference of exponentials filter which more closely resembles the antisymmetric STDP profiles found experimentally in other systems (e.g. [25]). The required bandpass characteristic could be implemented using both classes of filter (results for the difference of exponentials filter not shown). The iSTDP profiles consistent with the bandpass requirements were characterized by a narrow deep LTD dip at about the time the excitatory and inhibitory inputs coincide, balanced by a wider shallower LTP region (additional LTP and LTD lobes are possible, as in the exact bandpass sync function, but biologically implausible). Furthermore, the upper frequency limit of the bandpass is fixed by the LTD dip width (the narrower and deeper the dip the higher the frequency at which gain transfer is possible) and the low frequency limit is fixed by the LTP region width (the wider and shallower the LTP region, the lower the frequency at which transfer can take place).

Further details on the relation of correlational learning rules to spike-timing dependent plasticity are given in Gerstner and Kistler [26], Roberts and Bell [27] and Morrison et al. [28].

### Experimental Procedures

#### Animals and slice preparation

Experiments were performed on 250–300 µm coronal slices of the brainstem containing the rostral part of the MVN and the central stump of the VIIIth nerve from Lister Hooded rats aged P18–38 (young adult animals), except where the aim was to examine the effect of age when slices from animals aged P13–17 (juvenile animals, around the time of eye opening) were used. All procedures were approved by the Ethical Review Panel, University of Edinburgh, and were carried out in compliance with the UK Animals (Scientific Procedures) Act 1986 (project licence 6003334). Animals of either sex were decapitated under isofluorane anaesthesia, and the brains quickly removed into ice-cold modified aCSF (composition (mM): NaCl, 87; KCl, 1.2; HEPES, 10; glucose, 25; sucrose, 75; KH_2_PO_4_, 1.25; MgCl_2_, 7; CaCl_2_, 0.5, equilibrated with 100% oxygen, pH 7.3). Slices were cut using a Vibratome 3000 (Intracel, UK), transferred to aCSF (composition (mM): NaCl, 140; KCl, 2.5; HEPES, 10; glucose, 11; NaH_2_PO_4_, 1.2; MgCl_2_, 1.3; CaCl_2_, 2.4, equilibrated with 100% oxygen, pH 7.3) for 1 hour at 36°C, and then maintained at room temperature for at least a further hour before transfer to the recording chamber.

#### Electrophysiology

Slices were maintained in bath solution (aCSF containing 100 µM picrotoxin, superfused at 2 ml/min at 33°C and equilibrated with 100% oxygen) for at least 20 min before recording. A bipolar stimulating electrode was placed at the lateral border of the MVN, in the region of the root of the VIIIth nerve [29], [30], [31] to stimulate vestibular afferent fibres. MVN neurons were visualized using infra-red differential interference contrast microscopy (Olympus BX51W1, Japan). Whole-cell patch recordings were obtained using borosilicate glass electrodes with tip resistances of 5–8 MΩ when filled with internal solution (composition (mM): potassium gluconate, 145; HEPES, 5; EGTA, 0.1; MgCl_2_, 2; K_2_ATP, 5).

Data were recorded using an Axopatch 200B amplifier, sampled at 20 kHz and filtered at 5 kHz (in current clamp) or 2 kHz (voltage clamp). All neurons included here had input impedances >100 MOhm and spike heights of >+40 mV, and showed an early fast post-spike after-hyperpolarsation (AHP) followed by a delayed slow AHP (‘type B’ cells [15], [32]). EPSCs were evoked by the stimulation of the vestibular afferent axons in the dorso-lateral aspect of the slice (50–400 µA for 100–400 µsec) at 15 sec intervals, at a holding potential of −65 mV in voltage clamp, where the spontaneous spiking of these neurons was prevented. Previous studies have similarly stimulated vestibular afferents in slices [29], [30], [31], [33], though it cannot be excluded that additional intra-nuclear inputs were also activated by the electrical stimulus. Control recordings were made for 7–10 min to ensure EPSC amplitudes were stable. The mean amplitude of control EPSCs in this study was 264±17 pA and the mean latency was 2.06±0.08 msec (n = 52). In all experimental conditions latencies were unchanged after induction (2.03±0.07 msec, n = 52). Series resistance was monitored throughout the experiment. MVN neurons in which the EPSC amplitude was smaller than 100 pA, or varied by more than 20% during the pre-induction period, were not studied further. Neurons in which series resistance changed by more than 20% over the duration of the recordings (40–60 minutes) were also rejected.

Each recorded cell was tested with one of two alternative induction protocols applied in current clamp mode, with the membrane potential held at −65 mV. In the input-spike timing (iST) protocol a 20 msec hyperpolarizing current injection, mimicking the time-course of an inhibitory post-synaptic potential (IPSP) in the MVN neuron, was repeatedly paired with a single vestibular nerve stimulus which was applied at various times relative to the membrane hyperpolarisation (1000 presentations at 0.5 sec intervals; e.g. Fig 3A, B) [19], [34]. The hyperpolarizing pulse was of a sufficient amplitude to hyperpolarize membrane potential to −80 mV at its peak. The effects of altering the temporal relationship between the excitatory and inhibitory inputs were explored by systematically varying the timing of vestibular stimulation relative to the hyperpolarizing pulse.

**Figure 4:**
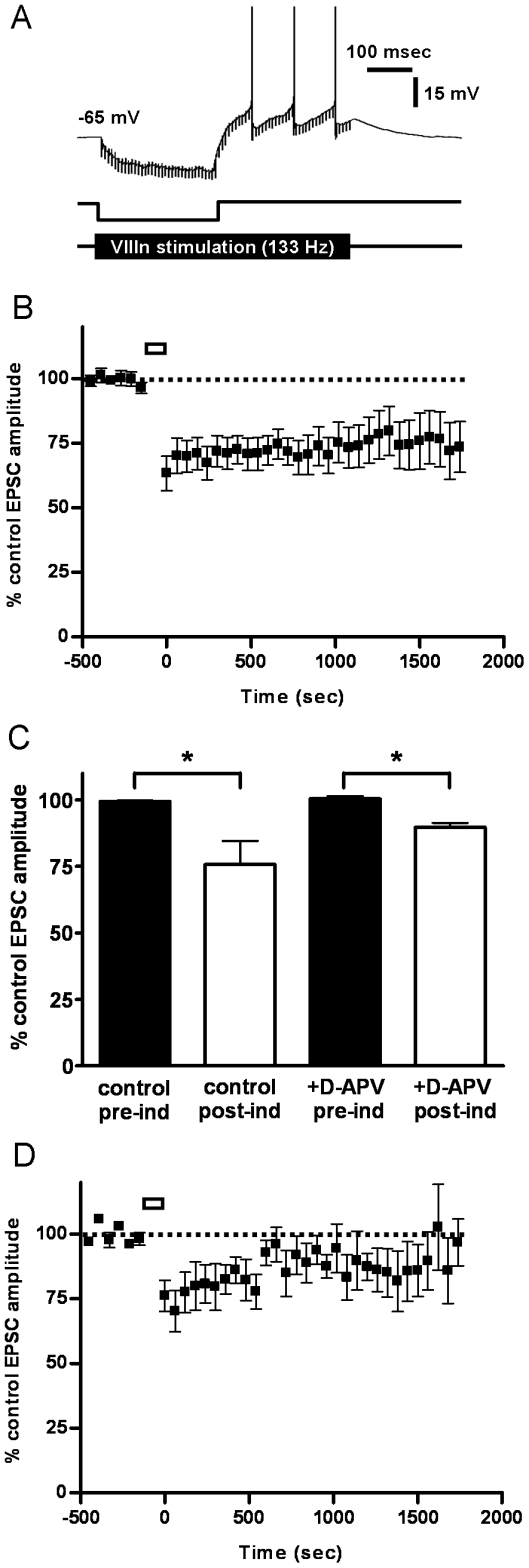
Plasticity of the vestibular afferent synapse induced with the pause-rebound protocol. A, Example of the pause-rebound (PR) induction protocol (after Pugh, 2006 #16]) in an MVN neuron. Stimulation of vestibular nerve at 133 Hz is accompanied by a hyperpolarizing current injection. Action potentials are truncated. B, Normalized EPSC amplitude before and after induction with the PR protocol (open bar) in MVN neurons from young adult animals aged P18 or older. EPSC amplitude is depressed significantly after PR induction (n = 8, p<0.03 compared to control). C, Pre-incubation with 50 µM D-APV did not prevent the induction of LTD in young adult MVN neurons using the PR protocol (controls, * p = 0.03, n = 8; +D-APV, * p = 0.01, n = 4). D, vestibular nerve stimulation alone, as in A but with the membrane potential held at −65 mV throughout, induced no lasting change in vestibular nerve EPSC amplitude (n = 4, p<0.36 compared to control).

In a number of MVN cells, the pause-rebound induction protocol devised by Pugh and Raman [19] to induce plasticity at the mossy fibre synapses on deep cerebellar nucleus (DCN) neurons (“PR protocol”), was used (e.g. Fig. 4A). In this case the vestibular afferents were stimulated at 130 Hz for 550 msec, and a hyperpolarizing current pulse (duration 250 msec, of a sufficient amplitude to hyperpolarize membrane potential to −80 mV) was injected coincident with the start of vestibular stimulation. This sequence was repeated 30 times every 5 seconds [19]. The total number of vestibular nerve stimuli presented in the iST and PR induction protocols are approximately equal. After either induction protocol, EPSCs were measured in voltage clamp mode with the membrane potential held at −65 mV for at least 30 min. At the end of some experiments, the vestibular stimulus-evoked inward currents were confirmed to be glutamatergic by abolition with 1 mM kynurenic acid.

Drugs used were picrotoxin, D(-)-2-amino-5-phosphonopentanoic acid (D-APV) and kynurenic acid (Sigma, UK).

#### Statistical Analyses

Data are presented as mean ± S.E.M. EPSC amplitudes (pA) were binned into 1 min periods, and normalised to the average EPSC amplitude in the pre-induction period. To determine the changes in EPSC amplitude after exposure to an induction protocol, a mean of 5 normalised bins recorded immediately prior to induction and a mean recorded 25 min post-induction were compared using a two-tailed paired t-test. To compare the effects of different induction protocols, a mean of 5 post-induction bins from each protocol were compared using a two-tailed unpaired t-test. The series of iST induction protocols was analysed by one-way ANOVA with Dunnett's multiple comparison post test with the Ts = −150 induction protocol designated as the control. P<0.05 was considered significant.

## Results

### Input-Spike Timing Dependent Plasticity

The term STDP does not by itself specify whether the relevant timing is between two sets of input spikes to a neuron, or between input spikes and postsynaptic action potentials. However, in practice it is so closely associated with the latter alternative that the term iSTDP (input-Spike Timing Dependent Plasticity) is used here for a proposed learning rule in which synaptic weight change depends on the relative timing of excitatory and inhibitory input spikes (see METHODS). The term “input timing dependent plasticity” (ITDP) has been used by Dudman et al [35] for the particular case of inputs to distal and proximal dendrites of hippocampal CA1 pyramidal neurons, but the term appears not to have been widely used subsequently.

#### Derivation of iSTDP Profiles

Theoretical analyses of VOR gain-adaptation have suggested a correlational learning rule for plasticity in the vestibular nuclei (Fig 1). However, this rule is typically expressed in terms of firing rates, whereas comparison with *in vitro* results requires the rule to be in input-spike-time dependent plasticity form. The derivation of this iSTDP form for the linear case is described in Methods, and the outcome illustrated in Fig 2.

The simplest version of the iSTDP rule is that if vestibular and cerebellar spikes arrive at an MVN neuron at the same time, substantial LTD is induced at the vestibular synapse. When the spikes arrive at different times, much weaker LTP is induced. The green traces in Fig 2 show the iSTDP profile and learning for the hypothetical case where the correlational learning rule applies uniformly for all frequencies of input (here, all frequencies of head velocity). In this case there is an infinitely narrow LTD dip at the time of coincidence of the two inputs, and an infinitely small LTP at all other times (Methods).

However, it is known that VOR adaptation learning is frequency dependent. At high frequencies learning-rate decreases above ∼2 Hz, reaching low levels at 10 Hz [11]. At low frequencies (<1 Hz) learning rates are confounded by the contribution of optokinetic and smooth pursuit systems: however, there are computational grounds for suggesting that learning at frequencies below ∼0.3 Hz should not be transferred to the brainstem [6]. These high and low frequency constraints are embodied in the blue curve of Fig 2B, and the iSTDP profile corresponding to them shown in Fig 2A. Comparison of blue and green profiles indicates that the general form of the iSTDP rule is preserved, although the LTD dip becomes broader and the LTP lobes narrower. The widening of the LTD dip is related to the high-frequency restriction on learning, with poorer high-frequency learning corresponding to a broader dip (see Methods for details).

The blue curve in Fig. 2A would predict the iSTDP profile for synaptic plasticity in the vestibular nuclei only if the frequency characteristics of VOR adaptation depended entirely on that plasticity. However, experimental evidence [11] shows that the high-frequency restriction is imposed by processing in the cerebellar cortex, and computational analysis [6] indicates that this cortical limit is a requirement for stable learning. In this case therefore the prediction is that the LTD dip for plasticity in the vestibular nuclei should be no wider than that shown in Fig. 2A, otherwise high-frequency VOR learning would be limited by vestibular, not cortical, processes.

#### Interactions between Vestibular EPSCs and Membrane Hyperpolarisation in MVN Neurons: Comparison with Predictions from the iSTDP rule

To test the above correlational rule experimentally, we explored the interaction between membrane hyperpolarisation and vestibular afferent EPSCs using the iST induction protocol consisting of a 20 msec hyperpolarizing current injection, mimicking the time-course of an inhibitory post-synaptic potential (IPSP) in the MVN neuron, paired with a single vestibular nerve stimulus that was applied at various times relative to the membrane hyperpolarisation (Fig. 3A, B; Methods). This protocol represents the simplest implementation of the above iSTDP learning rule.

Repeated pairing of the vestibular nerve stimulus with the onset of the hyperpolarizing current pulse (T_s_ = 0 msec, 1000 presentations at 0.5 sec intervals, Fig. 3A) induced a short-term depression of the vestibular afferent EPSC amplitude, which reversed within 15 minutes after the end of the induction protocol (Fig. 3C). By contrast, when the vestibular stimulus was timed to coincide with the end of the hyperpolarizing pulse, at the time when the membrane hyperpolarisation was at its peak (T_s_ = 20 msec, Fig. 3B), a robust long-term depression of the vestibular afferent EPSC amplitude was induced (Fig. 3D; mean normalised EPSC amplitude averaged between 25–30 minutes after induction was 63%±3% of pre-induction controls, n = 9, p<0.001).

There was no significant change in vestibular EPSC amplitude in slices pre-incubated with 50 µM D-APV for 15 minutes before the application of the induction protocol, indicating that the LTD was dependent on the activation of NMDA receptors (Fig. 3E; n = 4). Application of the vestibular stimulus at longer delays after the hyperpolarizing pulse (T_s_ = 40 or 60 msec), or in advance of the hyperpolarizing pulse (T_s_ = −150 msec) induced only short-term depression of EPSC amplitude, similar to that seen with T_s_ = 0 msec (Fig. 3F).

In three further MVN neurons, two induction protocols were applied in series: first the vestibular nerve stimulus was applied 150 msec in advance of the hyperpolarizing pulse (T_s_ = −150 msec) and then, after a observation period of 15 minutes post-induction, the vestibular nerve stimulus was applied at T_s_ = 20 msec (Fig. 3G). In these cells the first induction protocol induced a short-term depression of the EPSC amplitude which reversed within the 15 minute observation period, while the second induction protocol induced a marked and sustained LTD of the vestibular nerve EPSC (Fig. 3G; mean normalized EPSC amplitude after induction was 68%±2% of pre-induction controls, p<0.001).

Fig 3H compares an iSTDP profile that could partially fit the experimental data shown in Fig. 3F (red trace) with that derived from the frequency characteristics of behavioral VOR-adaptation learning *in vivo* (Fig 2, blue trace). The LTD dip found experimentally is consistent with the prediction above, being somewhat narrower than required by the *in vivo* VOR adaptation data (corresponding to better high-frequency learning in the range 5–20 Hz). The comparison shown in Fig 3H is thus consistent with a limit on high-frequency VOR learning imposed by processes in cerebellar cortex, rather than the brainstem [6], [10].

The theoretical iSTDP profiles show maximum LTD for an ISI 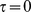, however we find maximum LTD when the vestibular stimulus coincides with the *end* of the 20ms membrane hyperpolarising pulse. This is presumably due to the biological constraints imposed by the underlying mechanisms, so that for example changes in membrane potential do not occur instantaneously following an inhibitory or excitatory input at 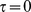, but instead follow a time-course determined by the membrane time-constant. While these results do not establish the precise timing for the iSTDP interactions around 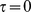, they show that the true timing error is likely to be less than 20ms. This corresponds to a Nyquist frequency of 25Hz; which is already close to the maximum frequency at which VOR gain is known to be adaptable.

### Comparison with Plasticity in Deep Cerebellar Nucleus (DCN) Neurons

#### Effects of the Pause-Rebound Protocol in MVN Neurons

The LTD obtained here with the iST protocol differs from the LTP found in experimental studies of DCN plasticity, albeit using a more complex protocol [19]. It was therefore important to test this apparent difference between MVN and DCN plasticity directly, by applying the complex protocol in the MVN. To compare the hyperpolarisation-dependent plasticity at the vestibular nerve synapse with that seen at the mossy fiber synapses in deep cerebellar nucleus (DCN) neurons, 13 further MVN neurons were tested using the PR induction protocol (Methods; after Pugh and Raman [19]; Fig. 4A). In mouse DCN neurons from animals aged P13–16, the PR protocol causes the activation of NMDA receptors and rebound firing, and induces LTP at the mossy fiber synapse [19].

In young adult MVN neurons (from animals aged P18–38) tested with the PR protocol (30 presentations every 5 sec), the amplitude of the vestibular nerve-evoked EPSC was significantly reduced compared to control and remained depressed for the duration of the recordings (Fig. 4B; mean normalised EPSC amplitude after induction was 76%±4% relative to pre-induction controls, p<0.03). In the presence of the selective NMDA receptor antagonist D-APV (50 µM), the PR protocol induced a smaller but still significant LTD of the vestibular EPSC (Fig. 4C). Vestibular nerve stimulation applied alone, without the concomitant hyperpolarizing pulse, induced only a short-lasting depression of the vestibular EPSC that reversed within 15 minutes post-induction, and LTD did not occur (Fig 4D; mean EPSC amplitude after induction was 94%±4% of pre-induction controls, p = 0.36).

#### Influence of Post-Natal Age

One possible explanation for the finding that the PR protocol induces depression of the vestibular synapses in MVN neurons, in contrast to the potentiation of mossy fibre synapses that occurs in DCN neurons [19], is that this reflects the differences in the post-natal ages of the animals used in the two studies. In rodents the eyes open for the first time at around post-natal day 15, and this is followed by a rapid visual system dependent maturation of the properties and synaptic function of vestibular nucleus neurons [15], [33], [36].

**Figure 5:**
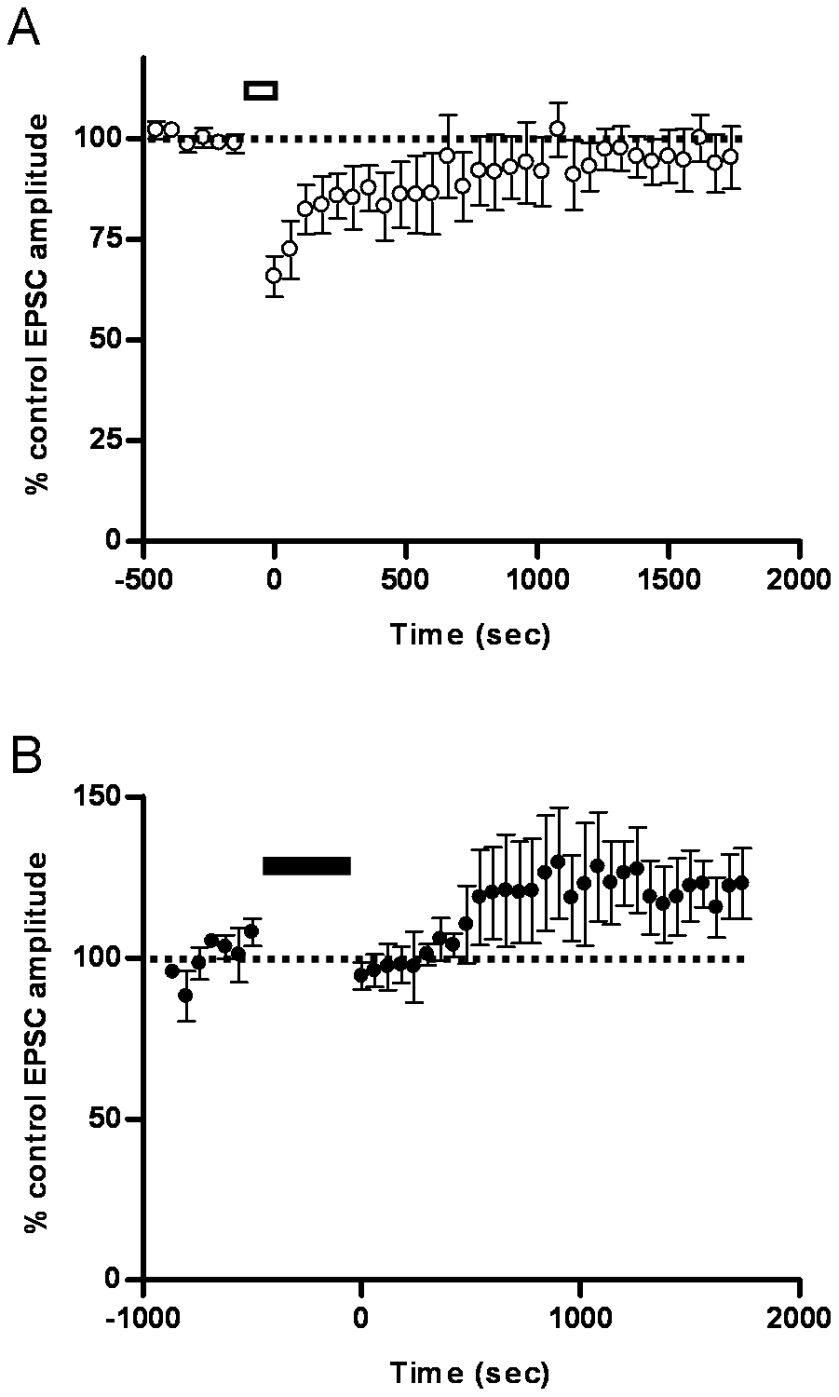
Effects of PR and iST induction protocols on plasticity at the vestibular afferent synapse in juvenile MVN neurons, in slices from animals aged P13–P17. A, Normalized EPSC amplitude before and after induction with the PR protocol in MVN neurons from juvenile animals aged P13–17 (n = 5; as in Fig. 4A, B). Note that in contrast to the hyperpolarisation-dependent LTD that occurs in young adult neurons (Fig. 4B), in juvenile neurons only a short-term depression of EPSC amplitude is observed. B, Normalized EPSC amplitude before and after induction with the T_s_ = 20 msec protocol in MVN neurons from juvenile animals aged P13–17 (n = 5; as in Fig. 3B, D). In MVN neurons from juvenile animals the iST protocol induces a delayed, but non-significant potentiation (21±4% potentiation; p = 0.1 compared to control).

Accordingly, we examined the role of post-natal age in two experiments. First, in juvenile MVN neurons recorded in slices from animals aged P13–17, the PR protocol did not induce LTD of the vestibular nerve EPSC but instead only a short-lasting depression of EPSC amplitude occurred (Fig. 5A). Secondly, we investigated whether the hyperpolarisation-dependent LTD of the vestibular afferent EPSCs evoked by the iST (T_s_ = 20 msec) induction protocol also occurred in juvenile MVN neurons. In contrast to young adult cells, in juvenile cells this protocol induced a small potentiation of the vestibular EPSC amplitude, which did not reach significance (Fig. 5B; mean normalised EPSC amplitude after induction was increased by 21%±4% relative to pre-induction controls, p = 0.1). Unlike the LTD of EPSC amplitude in young adult neurons, which was apparent immediately after the end of the induction and remained relatively unchanged for the following 30 minutes, the small potentiation in juvenile neurons developed gradually after a delay of some 10 minutes and increased to a plateau about 20 minutes post-induction (Fig. 5B).

#### Influence of Rebound

**Figure 6:**
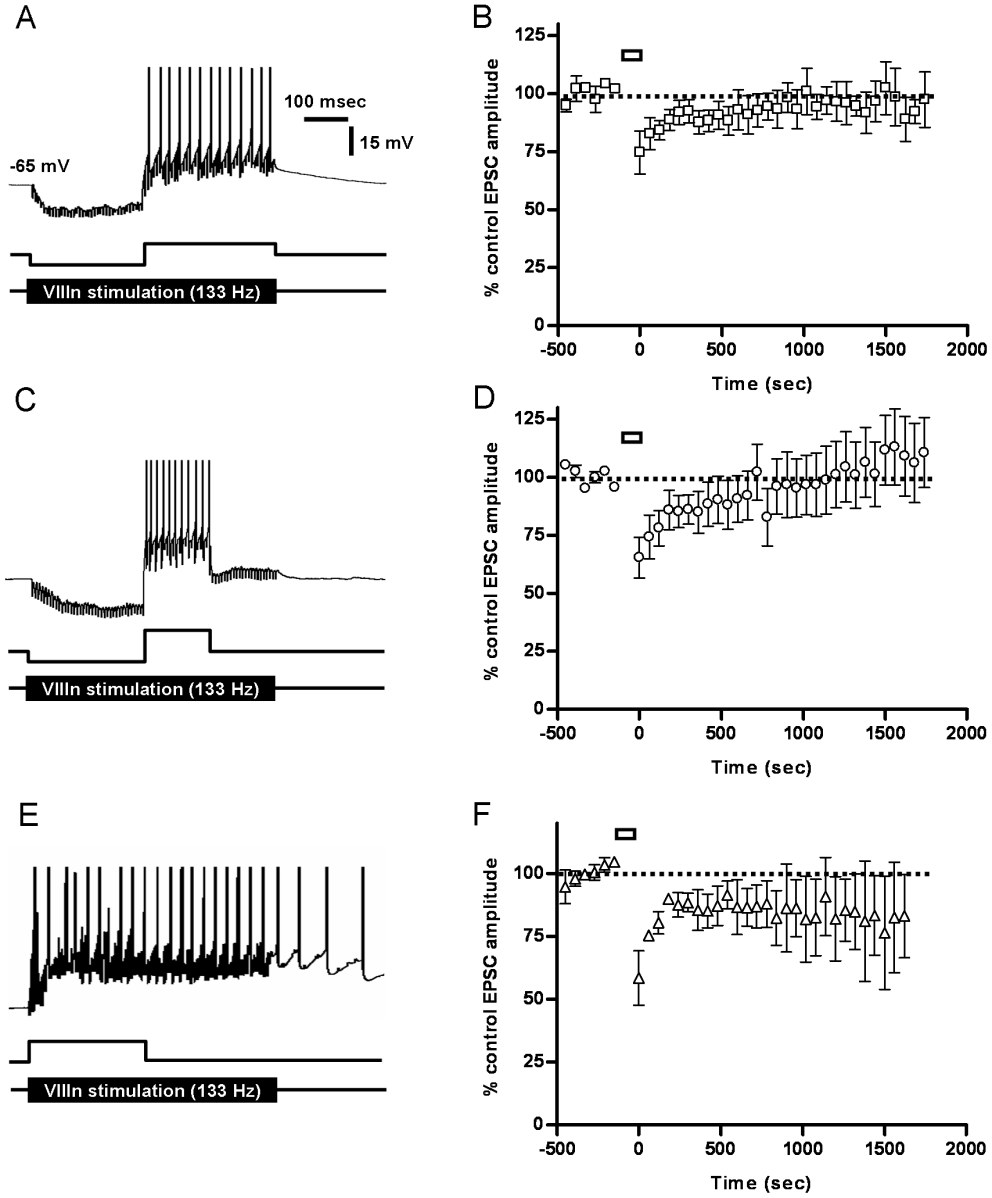
Post-inhibitory “rebound” depolarizing pulses occlude LTD at the vestibular afferent synapse induced with the PR protocol. A, Post-inhibitory membrane depolarizing pulses with a duration and amplitude equal to the inhibitory pulse (A, C) or with a duration of half that of the hyperpolarizing pulse but double its amplitude (B, D) occluded the expected LTD at the vestibular afferent synapse in response to the PR protocol, so that no long-term depression was induced (C, p = 0.6 compared to control, n = 4; D, p = 0.9 compared to control, n = 7). An alternative protocol where a depolarizing pulse was combined with vestibular nerve stimulation (E), also induced a short-term depression with no lasting significant change in EPSC amplitude (F; p = 0.2 compared to control, n = 3). Action potentials are truncated in A, C and E.

In mouse DCN neurons, LTP induced by the PR protocol depends upon mossy-fiber stimulation preceding a post-inhibitory rebound depolarization [19], [20], which may involve an influx of calcium into the post-synaptic cell through low-voltage-activated (LVA) calcium channels [19], [20], [37], [38], [39]. In the rat MVN, previous work has shown that relatively few neurons fire low-threshold Ca^2+^ spikes upon release from hyperpolarisation [13]. We therefore investigated the effects of mimicking a post-inhibitory rebound depolarization in young adult MVN neurons, using modified PR protocols where the membrane hyperpolarisation was followed by a depolarizing pulse which was either of the same amplitude and duration (Fig. 6A), or twice the amplitude and half the duration of the hyperpolarizing pulse (Fig. 6B). These modified protocols induced post-hyperpolarisation spiking at up to 70 Hz in the MVN neurons. Both of these protocols prevented the LTD of the vestibular nerve EPSC which was observed with the unmodified PR protocol (Fig. 6C, D cf. Fig. 4B).

Since the effect of the depolarizing pulses was to reverse the LTD induced with hyperpolarisation alone, we tested a further protocol consisting of a depolarizing pulse alone coinciding with the vestibular nerve EPSCs, to determine if this combination resulted in a discernible LTP at the vestibular synapse (Fig. 6E). However this protocol also induced only a short-term depression of EPSC amplitude, with no lasting change (Fig. 6E).

#### Modeling the Effects of the PR Induction Protocols on Synaptic Plasticity

**Figure 7:**
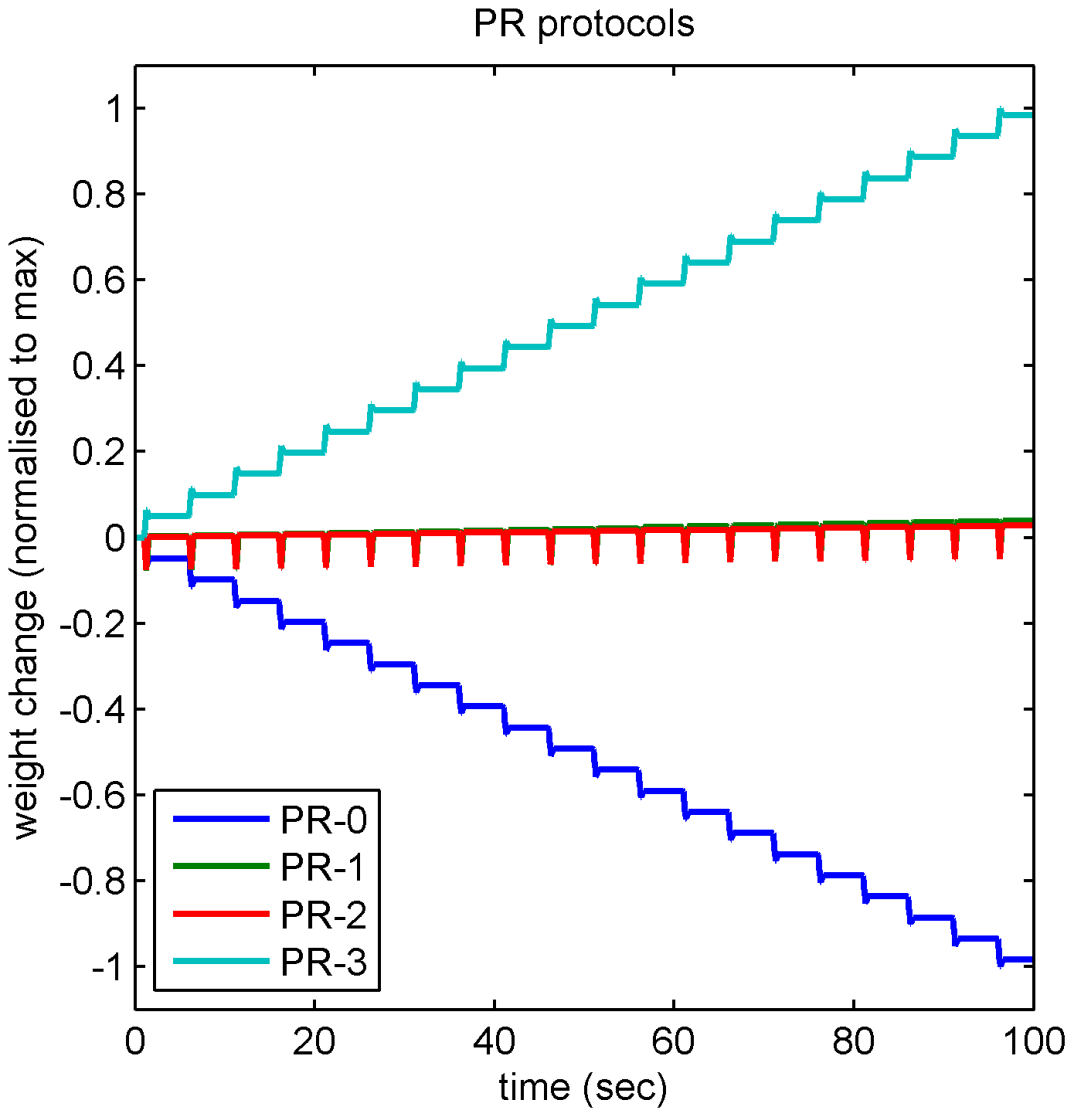
Modeling the effects of the original and modified PR protocols. The build up of weight change in vestibular synapses over time is shown for the three PR protocols. For PR-0 (a 250 msec hyperpolarization overlapping the 550 msec vestibular pulse train, see Fig. 4A) the main contribution at each presentation is LTD and there is a relatively large weight change. For PR-1 (250 msec hyperpolarization followed by 250 msec depolarization of equal amplitude) and PR-2 (250 msec hyperpolarization followed by 125 msec depolarization of twice the amplitude) the LTD contribution is approximately balanced by LTP leading to much smaller weight changes. For protocol PR-3 (a 250 msec depolarization overlapping the 550 msec vestibular pulse train, see Fig. 6F) overall LTP is predicted. In this simulation the hyperpolarizing and depolarizing current pulses were interpreted as due to proportional changes in firing rate of the appropriate input, and weight changes were calculated using the firing rate version of the learning rule (Equation 6).

To determine whether the effects of the PR protocols on vestibular synapse strength could be fully accounted for by the iSTDP learning rule, which is independent of any particular cellular mechanism, we modeled the changes in synaptic plasticity induced by the original and modified PR protocols. Inhibitory and excitatory inputs were treated as inputs to a linear system whose behavior was determined by its iSTDP profile (METHODS). Fig 7 indicates that this method successfully reproduces the marked cumulative LTD induced by the original PR protocol in vestibular neurons (PR-0, blue trace), as well as the substantial reduction in LTD when the protocols were modified as described above by addition of depolarizing pulses at the end of the membrane hyperpolarisation (PR-1 and 2, green and red traces).This pattern of results reflects the fact that during induction with the PR protocols most of the individual vestibular EPSCs are paired with membrane hyperpolarisation in the original protocol PR-0, whereas in the modified PR protocol the tendency is to produce nearly balanced LTD and LTP at each presentation since the EPSCs now coincide nearly equally either with membrane hyperpolarisation in the early part of the presentation or membrane depolarization in the later part. Since the modeling results essentially derive from the linearity of the modeled system, the implication is that this form of vestibular nucleus plasticity also behaves approximately linearly in the conditions studied here (cf. [40]).

A significant feature of this result is the dependence of the iSTDP learning rule upon the local membrane potential of the post-synaptic cell at the time of arrival of the EPSCs, not upon action potential firing of the post-synaptic cell. However, since the post-synaptic cell depolarization in the modified PR protocols is accompanied by spiking, this raises the possibility that post-synaptic spikes may also affect plasticity at the vestibular synapse. The PR protocols are ambiguous in this regard, since they necessarily confound post-synaptic depolarization with post-synaptic spiking. Resolving this confound experimentally, to determine whether post-synaptic spike firing may also have a role in regulating vestibular synapse strength, is an important issue that is difficult to address directly. This is particularly the case if the iSTDP interactions between excitatory and inhibitory inputs take place in the distal dendrites of MVN cells, some distance removed from the soma (Discussion). In the comparatively much better studied models of homosynaptic STDP in cortical neurons the issue of postsynaptic spiking versus slower membrane potential changes remains unclear. One possibility is that dendritic action potentials are simply too brief to engage the plasticity mechanism [41].

**Figure 8:**
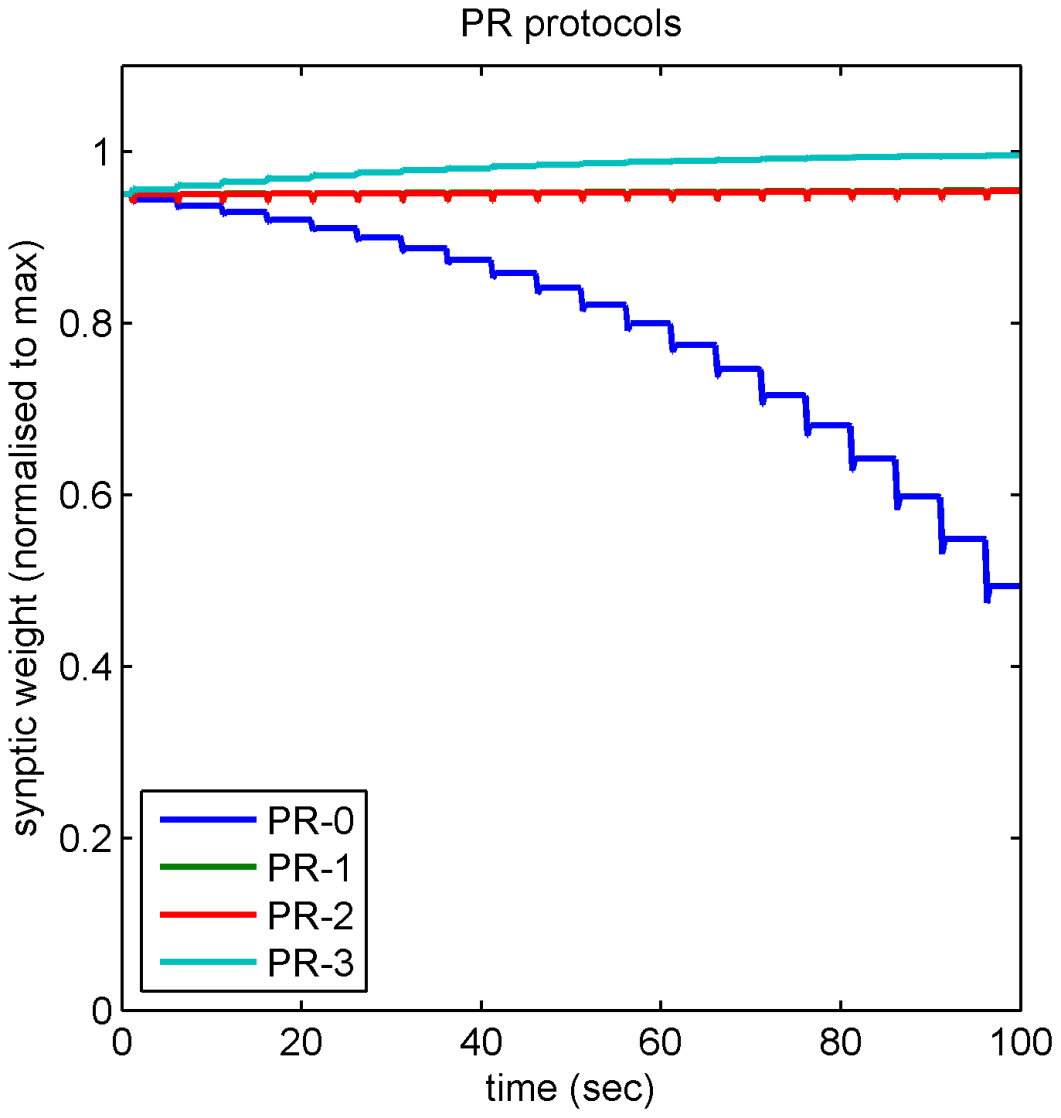
Effect of weight saturation on predictions for the PR protocols. Predictions for the weight changes that occur under the PR protocols shown in Fig. 7 are modified if the basic iSTDP learning rule is altered to include a model of weight saturation. Here weights have been normalised to a maximum of 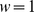, and the initial weight taken to be 95% of the maximum value. Normalised weight values are plotted (rather than relative weight changes as in Fig. 7) for clarity of interpretation. At each time step the weight change 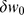 predicted by the basic iSTDP rule (equation (3)) and shown in Fig. 7, has been replaced by the new rule 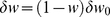. This rule implements a standard ‘soft’ saturation model (Gerstner and Kistler (2002), p. 385). The learning rate was chosen so that the LTD protocol PR-0 reduces the synaptic weight to 50% of its maximum value.

Our iSTDP model features both LTP and LTD, and its success in predicting the effects of the complex PR protocols in MVN neurons depend upon both. However in these experiments the LTP for these protocols is not observed directly, but is ‘implicit’ in the model. Nevertheless the linear model predicts explicit LTP for protocol PR-3 (Fig. 6F), raising the question of why the modified PR protocols show implicit but not explicit LTP. One possibility is that synaptic weights *in vitro* may be close to their maximum values, which would create a nonlinearity and mean that *in vitro* LTP could only be elicited after prior LTD. This possibility is shown in simulation in Fig. 8. Such a saturation of vestibular synaptic weights in the *in vitro* experimental conditions is actually predicted by the iSTDP learning rule because of the absence of cerebellar inhibitory synaptic inputs to the MVN neurons in the slice preparation. Thus while *in vivo* the ongoing simple-spike firing of Purkinje cells would provide a continuing inhibitory synaptic input to the MVN neurons, inducing a steady level of LTD in the vestibular synapses, the lack of this inhibitory input *in vitro* may allow the vestibular synapse strength to drift towards the maximal value.

### Modeling Applicability of iSTDP to *In Vivo* Conditions

Since the nature of the plasticity induced by the iSTDP rule depends upon accurate spike timing, it is important to show that it is equivalent to a rate-coded learning rule for the stochastic, asynchronous spiking inputs received by MVN neurons *in vivo* [42], [43]. It is therefore important to investigate in simulation whether the proposed rule would be effective for spontaneously firing MVN neurons receiving realistic inputs.

**Figure 9:**
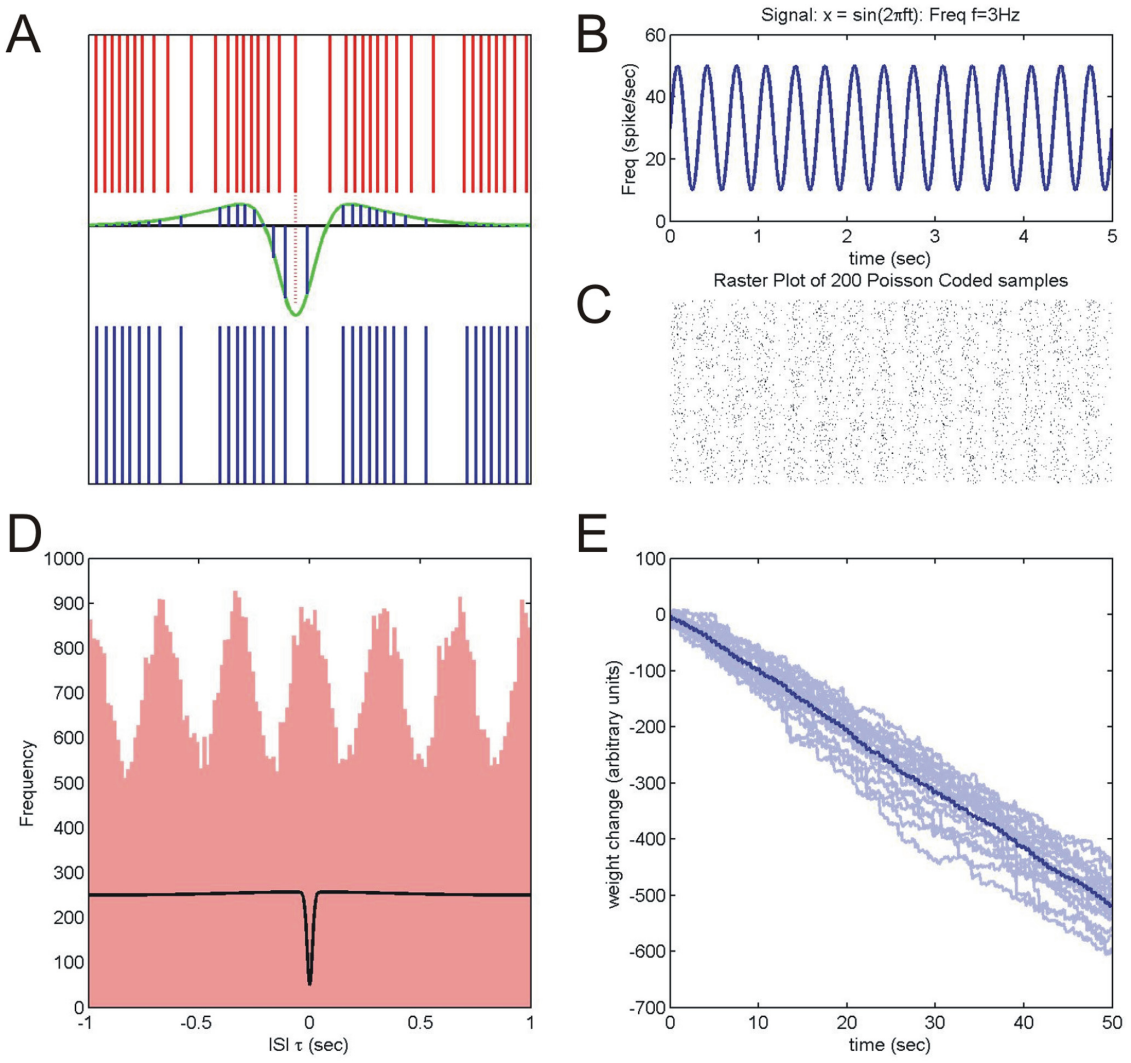
iSTDP for stochastic inputs. **A:** The top (red) spike train represents vestibular input 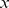 and the bottom (blue) spike train represents inhibitory cerebellar input 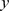. The green curve shows an iSTDP profile (corresponding to a difference of Gaussians kernel 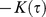 as described in Methods) chosen to demonstrate both LTP and LTD lobes clearly. The total contribution of a given vestibular spike, for example the one extended by the red dotted line, to synaptic weight change is calculated as follows: its timings 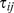 with respect to all cerebellar spikes are determined. The contribution of each of these spike pairs to weight change is proportional to 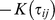 (equation (3)), these values are shown graphically as the blue segments under the iSTDP profile centered at the chosen vestibular spike. The sum of all these segments is the weight change ‘caused’ by that vestibular spike. **B:** A sinusoidal vestibular input modulating at 3 Hz, represented as a variation in firing rate with mean rate 30 Hz and amplitude 20 Hz. **C:** A raster plot of 200 different Poisson coded samples of the sinusoidal signal (for Poisson coding the probability of a spike in a short interval 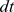 is 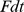 where 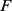 is the firing rate to be coded). It is clear that the coding scheme is asynchronous, i.e. the timing of individual spikes is not well-determined. **D:** A histogram of all interspike intervals τ (between vestibular and cerebellar spikes, see Methods) for a single 50 s 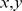 input pair modulated in phase at 3 Hz and Poisson coded as in Fig. 2. Despite the fact that individual spikes are not precisely timed, there is a clear modulation of the ISI histogram at 3 Hz with a peak at zero ISI. The experimentally constrained iSTDP profile from Fig. 3H is overlaid on the histogram. **E:** Cumulative weight change calculated for 20 pairs of 50 s samples of vestibular and cerebellar input. There is a stochastic but consistent weight decrease. The mean weight change (dark blue curve) is also shown.

Fig 9A illustrates the operation of the iSTDP rule (equation (3), Methods). Fig. 9B shows a sinusoidally modulated input signal and below it (Fig. 9C) a raster plot of multiple spike train samples generated from it using a stochastic (Poisson) model. It is clear that this coding procedure is asynchronous, i.e. that the timing of individual spikes is not well-determined, so that for sinusoidal inputs generated in this way we cannot guarantee the accurate relative spike timing between vestibular and cerebellar input spikes that seems to be required by the iSTDP algorithm.

However Fig. 9D shows a histogram of all ISIs between spikes in the two input streams for two sample 50 s segments. Although individual absolute spike timings are not well defined, it is clear that the correlated frequency modulation between the spike trains is accurately reflected in the statistical properties of the ISIs. In particular the positive correlation leads to an excess of spike pairs with small ISIs; these produce net LTD because they lie in the narrow LTD dip in the iSTDP profile (shown superimposed). Finally Fig. 9E shows the cumulative weight changes for several 50 s samples of positively correlated sinusoidal inputs. Although the weight changes show some stochastic variation there is a consistent steady decrease in synaptic weight as predicted by theory. As with the PR protocol simulations shown in Fig. 7, the findings shown here assume linearity of the modeled system [26].

The fact that these results illustrate how the model can work well in principle points to the necessity for further experimental work, to establish whether the implicit LTP or LTD reduction observed here is in fact driven by membrane depolarization as predicted by the model, and to clarify the role of post-synaptic spiking. The assumption of linearity used here, which means that the iSTDP profile against a null background also applies at other firing rates, is widely made in spike-timing dependent plasticity models [26], [44] yet is not often tested empirically. Determining the cellular mechanisms involved in implementing the iSTDP rule is therefore important for understanding vestibular neuron plasticity, and may also contribute to understanding plasticity in other systems. For example, although conventional STDP in cortical neurons has been extensively studied since its initial description [45], the underlying cellular mechanisms are still a matter for current debate [41].

### Robustness of Modeling Results

The robustness of the modelling results shown in Fig. 9 was investigated in three ways. Firstly they were shown to be robust with respect to the shape of iSTDP profile (symmetrical *versus* asymmetrical, see Methods).

**Figure 10:**
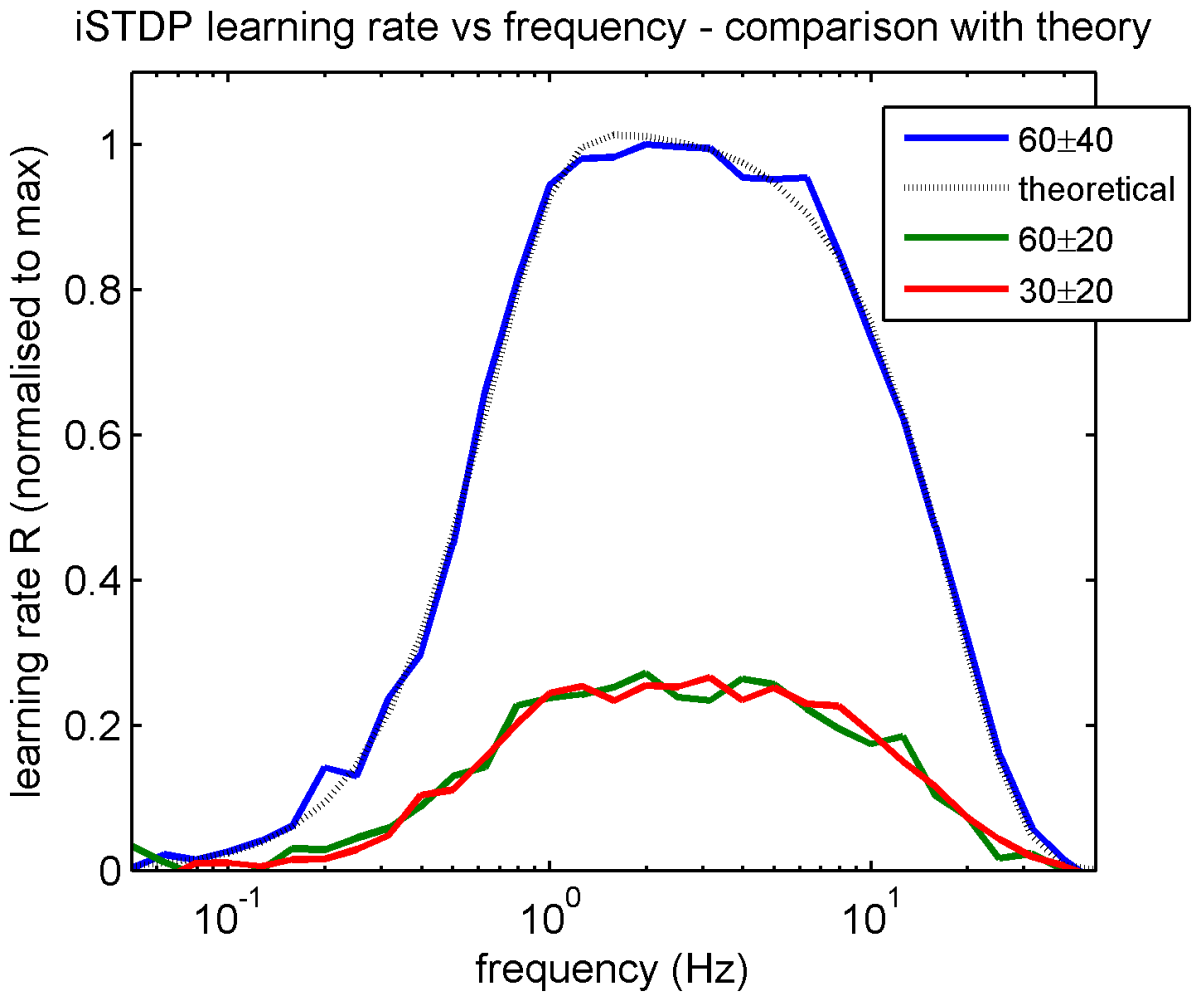
Robustness of iSTDP learning rule. The robustness of the iSTDP learning rule for stochastic spiking inputs was checked by comparing the learning rates of spiking simulations with the theoretical predictions from equation 7. Effective learning rate is plotted as a function of frequency for the iSTDP profile constrained by the experimental data shown in Fig. 3H. Learning rate was calculated for sinusoidally modulated Poisson spike trains using the procedure described in Fig. 8 for in-phase sinusoidal modulation frequencies in the range 0.05 to 50 Hz. The blue curve is for spike trains with tonic rate 60 spikes/sec and a modulation depth of 40 spikes (summarised as 60±40). The theoretical (dashed) learning rate curve 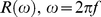 (also shown in Fig. 2B) is overlaid for comparison. The green curve (60±20) and red (30±20) learning rate curves are calculated using different values for tonic and for both tonic and peak modulation firing rates respectively. The results illustrate the theoretical predictions that (i) peak learning rate is proportional to the product of modulation rates (in this case both peak modulations are halved, reducing peak learning rate to 25% of its value) (ii) the overall shape (frequency dependence) of the learning curve is unaffected by modulation level, and (iii) the tonic rate has no effect on learning.

Secondly the equivalence of the spiking and firing rate formulations of the learning rule was checked. The frequency and depth of input modulation, and baseline tonic firing rate of stochastic firing rate coded signals were varied (Fig 10). The effects of frequency on learning rate for these spiking simulations correspond to those predicted theoretically by equation (7). Similarly in accord with theoretical predictions, peak learning rate is proportional to the product of modulation rates, the overall shape (frequency dependence) of the learning curve is unaffected by modulation level, and tonic rate has no effect on learning.

**Figure 11:**
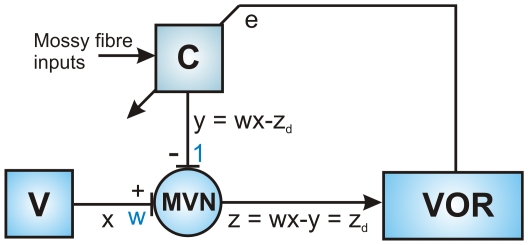
Effect of LTP/LTD imbalance: Modeling brainstem learning in the closed loop. The vestibular and cerebellar input signals 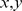 are coded stochastically as modulations of tonic firing rates 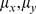. When LTP exactly balances LTD the contribution to weight changes from the mean firing rates is zero. Any imbalance however produces an additional term in the learning rule, proportional to the product of the mean firing rates. In the open loop (i.e. when VOR inaccuracy has no effect on the system) this term would tend to drive the weight 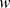 to saturation (for an excess of LTP) or to silence (for an excess of LTD). Hence any LTP/LTD imbalance, however small, potentially leads to learning instability. This situation is modified when the behavioural closed-loop via the cerebellum is considered. Whereas error in 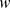 is generated at the slow time-scale of brainstem learning, the retinal slip 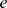 that the weight error entails drives cerebellar cortical learning on a much faster time scale, modifying the cerebellar input 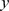 to the MVN so as to correct the error. We will show that this modification tends to stabilise learning at the MVN synapse (Fig 12). The cerebellar module C learns to compute its output 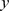 from inputs on its mossy fibres (vestibular signals and motor efferent copy signals) guided by the retinal slip teaching signal 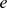, To allow efficient simulation of the closed loop situation we assume that cerebellar cortical learning is accurate and much faster than brainstem learning so that the cerebellar input is always optimal for the current synaptic weight 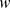, that is,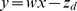 where 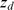 is desired MVN output. If the desired output of the MVN neuron is 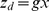, i.e. we have a target overall gain at this stage of 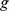, then 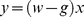.

Thirdly, the effects of unbalanced iSTDP profiles were examined. An unbalanced profile is one in which the area under the LTD part of the profile is not equal to that under the LTP part. In the simplest case, where the inputs to the vestibular nucleus are unaffected by changes to its output, the main effect of an imbalance is that tonic firing rates produce a constant rate of weight change, eventually driving the synaptic weight to its upper or lower limit (results not shown: cf. [46]). However, the vestibular nucleus is in fact part of a closed loop together with cerebellar cortex (Fig. 11), which confers some degree of internal stability. The gain errors caused by LTP/LTD imbalance produce retinal slip, which drives cerebellar learning in a direction so as to cancel the gain error. As a result the cerebellar input contains a component which is correlated with vestibular signal. This correlation drives gain transfer in a direction opposing the effect of the original LTP/LTD imbalance.

**Figure 12:**
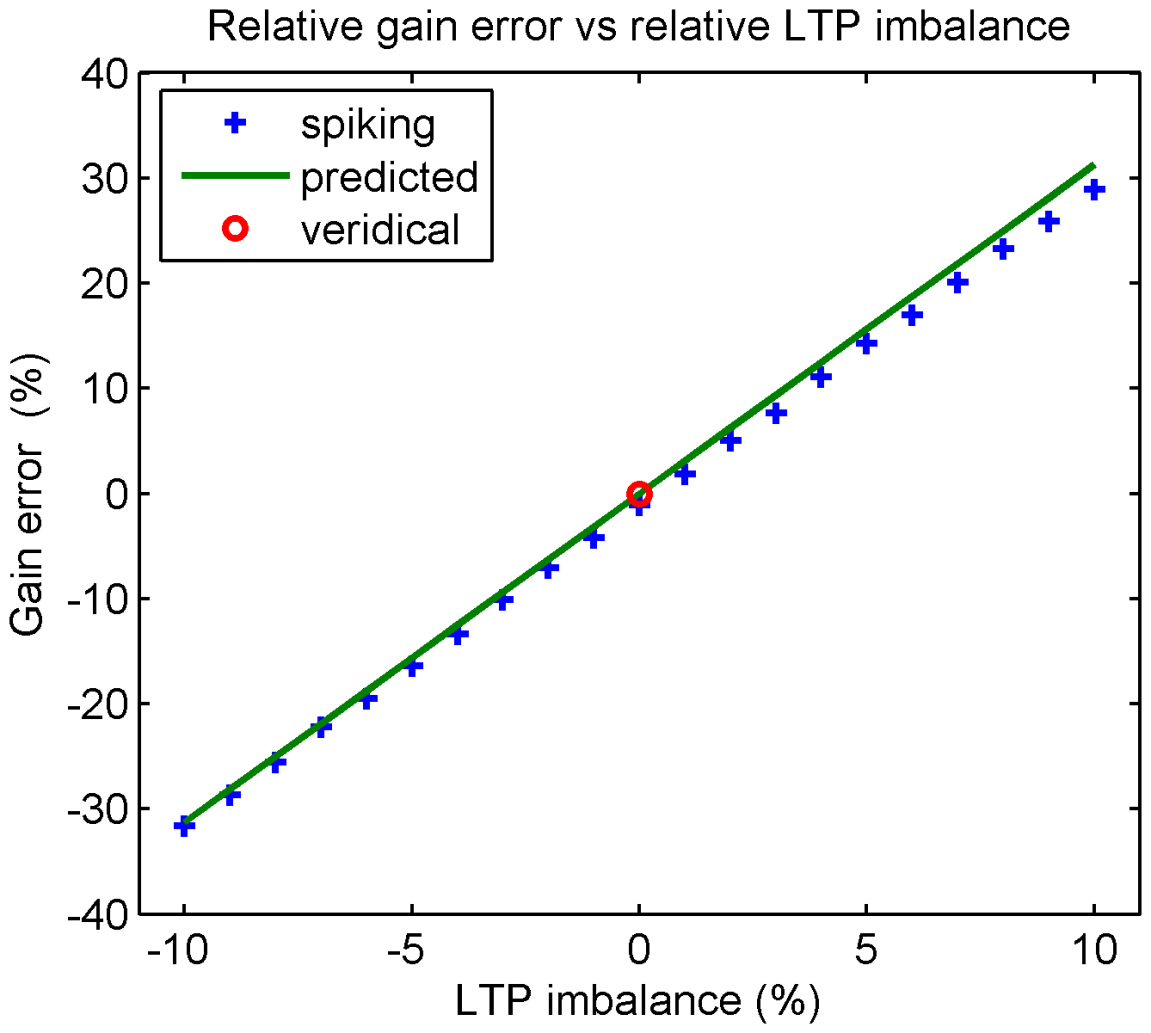
Effect of LTP/LTD imbalance: Stability of brainstem learning rule in the closed loop. The blue crosses show the result of applying the iSTDP learning rule with varying levels of LTP/LTD imbalance to an MVN neuron with a unit amplitude 3 Hz sinusoidal vestibular input 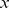. As outlined in Fig. 10 the corrective cerebellar input was set to 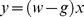 as 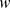 varied during learning. Both 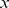 and 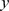 were Poisson rate-coded with tonic rates of 50 spikes/sec and a modulation depth of 40 spikes/sec. LTP imbalance was measured as the percentage excess of the area of the positive Gaussian over the negative Gaussian in the iSTDP profile (other iSTDP profile parameters were chosen as in Fig. 8). The target gain was 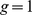. For moderate levels of imbalance learning produced a stable limiting weight 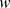. For zero imbalance the target gain is all expressed in the MVN weight 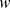, hence we used 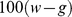 as a measure of the percentage weight error caused by the imbalance. This is also the percentage VOR gain error that would be observed if the cerebellar contribution was removed. The observed linear dependence is well predicted by the idealised iSTDP profile shown in Fig. 2 (green curve) for which the learned weight can be calculated analytically as 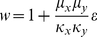 where 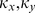 are the peak modulations of the two inputs and 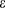 is the relative imbalance in LTP. Weight error calculated using this approximation is plotted as a green line.

Simulations of this behaviour (Fig. 12) show that the effect of imbalance is to produce errors in the gain transferred to the brainstem, but not weight divergence, as long as the imbalance is relatively small. However, a relative imbalance of ±10% leads to gain transfer errors of ±30%. Thus a reasonably accurate transfer of gain to the brainstem is only possible if the LTD/LTP imbalance is small. These results therefore suggest that there may be homeostatic cellular mechanisms that balance LTP and LTD over the longer timescale [47], which would be appropriate to maintain stability in a system with tonic firing rates.

## Discussion

These findings demonstrate a novel form of plasticity in vestibular neurons, in which a robust LTD develops at the vestibular synapse when afferent EPSPs occur simultaneously with membrane hyperpolarisation, intended to simulate IPSPs evoked by cerebellar inhibitory inputs. The development of LTD requires a precise coincidence of EPSPs with membrane hyperpolarisation, so that a large LTD occurs when the vestibular stimulus coincides with the peak of the simulated IPSPs while weaker or no LTD results from EPSCs occurring before or after (Fig. 3). This result agrees with predictions from an iSTDP implementation of the correlational learning rule for cerebellum-guided plasticity in the vestibular nuclei (Fig 2) [4], [5], [6], [12], [48], and also with the experimental demonstration that VOR adaptation [4], [6] can be driven by simple-spike instructive signals [49].

In the present experiments, membrane hyperpolarizing currents were injected via the recording electrode on the MVN cell soma in order to induce LTD at the vestibular synapses. In reality, Purkinje synaptic terminals may primarily influence vestibular afferent synapses in their immediate vicinity. Convergence of vestibular afferents and Purkinje terminals onto individual dendritic compartments [50], [51], [52], [53] could enable a highly effective, localized implementation of the iSTDP mechanism. Indeed, in a realistic model of an MVN cell dendrite, EPSCs coinciding with membrane hyperpolarisation induce elevations of sub-synaptic [Ca^2+^] broadly consistent with the LTD observed here (Graham, Menzies and Dutia, unpublished results).

### iSDTP at the Vestibular Afferent Synapse

The iSTDP profiles for vestibular synaptic plasticity resemble the anti-Hebbian STDP profiles [27] observed in Purkinje-like cells of mormyrid electrosensory lobe [54] and spiny stellate cells of cerebral cortex [55]. Here however the learning rule is heterosynaptic, with plasticity depending upon the relative timing of excitatory and inhibitory synaptic inputs, instead of homosynaptic, depending upon the timing of excitatory inputs and post-synaptic action potentials. This independence from post-synaptic firing distinguishes heterosynaptic iSTDP from proposed mechanisms for plasticity at mossy-fiber synapses on DCN neurons, that require a period of cerebellar inhibition of the DCN neurons sufficient to silence them, followed by a rebound depolarization and calcium influx [19], [20]. For VOR gain adaptation any requirement for silencing of the post-synaptic neurons is problematic, since MVN neurons are themselves directly involved in gaze holding and VOR execution. If vestibular synaptic strength is regulated through iSTDP interactions in distal dendritic compartments, as suggested above, this may be potentially dissociated from somatic spiking. The electrophysiological properties of MVN cell dendrites, and the extent to which somatic spikes propagate antidromically, are presently unknown. While the precise cellular mechanisms remain to be investigated, the independence of the iSTDP learning rule from post-synaptic spiking offers a potential solution to this difficulty.

A further advantage of the iSTDP learning rule is that it successfully predicts the effects of more complex induction protocols on vestibular synaptic plasticity (Figs. 4, 6 and 7). The PR protocol [19], [20] designed to mimic the pattern of inputs to DCN neurons presumed to occur during eyeblink conditioning [56], is substantially more complex than the pairing of single EPSCs and simulated IPSCs which represents the simplest implementation of the correlational rule. Predictions from modeling of iSTDP interactions between EPSCs and membrane hyperpolarisation closely match the effects on vestibular neurons of both the PR induction protocol, and of variants with post-pulse depolarizations. This suggests that iSTDP interactions may be generally applicable and predict the modulation of vestibular synapse strength by convergent inputs under a variety of conditions.

Unexpectedly, we did not observe LTP that was predicted to occur at conjoint stimulation delays outside the time window that produces LTD (Fig 2). Since the predicted LTP is always much smaller than the observed LTD, it is possible that the protocols used here were not sensitive enough to detect it. However, we were also unable to demonstrate explicit LTP with a more complex protocol specifically designed to induce it (Figs. 6E, 7), though this result needs confirming with a larger number of observations. It is unlikely that the vestibular synapse is incapable of expressing LTP, since even a slight imbalance between LTD and LTP would lead to either to weight saturation (cf. [46]), or at best to a degraded VOR gain transfer (Fig. 11). Moreover, the effects of protocols PR-0,1 and 2 were well predicted by an iSTDP rule that assumed balanced LTD and LTP, such that EPSPs coinciding with membrane depolarization tended to counteract the LTD induced by EPSPs coinciding with membrane hyperpolarisation. One way in which this implicit LTP could be reconciled with absence of explicit LTP is if the synaptic weights *in vitro* were close to their maximum values (Fig. 12). This possibility requires further experimental investigation, for example by first reducing the weights with protocol PR-0 prior to testing for LTP with protocol PR-3.

In addition, further work is necessary to determine if the proposed iSTDP mechanism is present specifically in flocculus target neurons (FTNs). In the rodent MVN, only a proportion (∼5–20%) of neurons are FTNs [34], [57], [58], and recent evidence indicates that different MVN cell types have importantly different properties (e.g. [59], [60], [61], [62]). Although the protocols used here did not indicate any qualitative differences with respect to LTD induction, the issue requires more systematic investigation.

While specific iSTDP interactions between vestibular and Purkinje cell inputs may regulate vestibular synapse strength in the context of VOR gain adaptation, these results also raise the possibility of interactions with other, non-cerebellar inhibitory inputs that induce membrane hyperpolarisation co-incident with vestibular EPSCs. Indeed recent work has shown that inhibitory interneurons activated by vestibular afferents provide an important feed-forward inhibition to second-order vestibular neurons, particularly type B cells [63]. In contrast to the cerebellar inhibitory inputs however, the feed-forward inhibition is not thought to be modulated by eye-movement signals and so is unlikely to be involved in VOR gain adaptation. Instead, it may be hypothesized that iSTDP interactions with feed-forward inhibitory inputs could induce a steady, activity dependent homeostatic LTD at the vestibular synapses. This is consistent with our suggestion that in the absence of such inputs *in vitro*, vestibular synapses may drift to be close to their maximal strength in slices (Fig. 8). The role of such possible interactions between vestibular and non-cerebellar inhibitory inputs remains to be determined.

### Relation to Plasticity in Deep Cerebellar Nuclei

The different effects of the PR protocol on MVN and DCN neurons may reflect differences in post-inhibitory rebound firing between the two cell types, perhaps due to differences in expression of LVA Ca^2+^ channels. However, recent evidence suggests that rebound burst firing in DCN neurons may not occur *in vivo* [64], [65]. The present results suggest the alternative possibility that iSTDP interactions could also modulate the strength of mossy-fiber synapses on DCN neurons in physiological conditions. If so, consistent pairing of a *reduction* in inhibitory input with an increase in mossy-fiber firing would cause LTP of mossy-fiber synapses. This has been proposed as a learning rule in a model of eyeblink conditioning (equation 6 in [66]; see also [56]), and is consistent with the striking pause in Purkinje cell firing that occurs during conditioning [67]. Whether mossy-fiber firing in the relevant part of the DCN does increase during eyeblink conditioning is yet to be established [68].

### Cellular Mechanisms

Input-spike-timing dependent LTD at the vestibular synapse is prevented by the NMDA receptor antagonist D-APV, indicating that it requires NMDA receptor activation and Ca^2+^ influx at relatively hyperpolarized membrane potentials. In addition however, modeling of the interaction between membrane potential and vestibular EPSCs in an MVN cell dendrite (Graham, Menzies and Dutia, unpublished results) indicates activation of both NMDA receptors and low-voltage activated Ca^2+^ channels, so that Ca^2+^ influx from both sources is likely to be necessary for LTD. NMDA currents are active around the resting potential in MVN cells, and contribute to their resting discharge and the vestibular nerve EPSC [29], [69], [70], [71]. Indeed a form of NMDA-receptor dependent LTD, induced by high-frequency stimulation of vestibular afferents *in vitro*, has been reported by Grassi et al [72]. Consistent with this, mRNA and protein for NR2C and NR2D NMDA receptor subunits have been demonstrated in vestibular neurons [73], [74], [75]. When incorporated with NR1 subunits, these subunits confer a low sensitivity to the NMDA receptor to Mg^2+^ block and allow significant inward Ca^2+^ current even at relatively hyperpolarized membrane potentials [76], [77]. Furthermore, NR2C subunit expression in MVN neurons appears between P7 and P10 and increases to reach adult values after P21 [75]. This is in line with our observation that LTD of the vestibular EPSC does not occur in juvenile MVN neurons, but is only seen in young adult cells (Fig. 4, 5). Thus iSTDP-dependent LTD requires the post-natal maturation of NMDA receptor expression after eye-opening, in a similar way to the vision-dependent effects on plasticity at the vestibular nerve synapse observed by Grassi et al. [33]. In juveniles therefore the naïve system appears to favor the maintenance of vestibular synapses, while the correlative rule for experience-dependent adjustment of vestibular synapse strength develops only after eye-opening. The cellular mechanisms involved in the implementation of the iSTDP rule remain to be elucidated.

In conclusion, these results suggest the cerebellum alters the strength of vestibular synapses on MVN neurons through hetero-synaptic, anti-Hebbian iSTDP. The iSTDP rule predicts the LTD of vestibular synapses when excitatory and inhibitory inputs interact within a precise temporal window, and also predicts the effects of more complex trains of excitatory and inhibitory inputs. Since the iSTDP rule does not depend on post-synaptic firing, it suggests a possible mechanism for VOR adaptation without compromising gaze-holding and VOR performance *in vivo*.
